# Pharmacokinetic Landscape and Interaction Potential of SGLT2 Inhibitors: Bridging In Vitro Findings and Clinical Implications

**DOI:** 10.3390/pharmaceutics17121604

**Published:** 2025-12-12

**Authors:** Nahyun Koo, Eun Ji Lee, Ji-Eun Chang, Kyeong-Ryoon Lee, Yoon-Jee Chae

**Affiliations:** 1 College of Pharmacy, Woosuk University, Wanju 55338, Republic of Korea; 2 College of Pharmacy, Dongduk Women’s University, Seoul 02748, Republic of Korea; 3 Laboratory Animal Resource Center, Korea Research Institute of Bioscience and Biotechnology, Cheongju 28116, Republic of Korea; 4 Department of Biotechnology, University of Science and Technology, Daejeon 34113, Republic of Korea; 5 Research Institute of Pharmaceutical Sciences, Woosuk University, Wanju 55338, Republic of Korea

**Keywords:** SGLT2 inhibitors, drug interactions, pharmacokinetics, type 2 diabetes mellitus, polypharmacy

## Abstract

Sodium–glucose cotransporter 2 (SGLT2) inhibitors are widely used in type 2 diabetes and cardiometabolic diseases, and their pharmacokinetic characteristics generally confer a low risk of clinically relevant drug–drug interactions (DDIs). Most clinical studies demonstrate that these agents can be co-administered safely with commonly prescribed medications without dose adjustment, although strong enzyme inducers such as rifampin can reduce systemic exposure, and pharmacodynamic interactions may still arise. However, existing evidence is largely derived from short-term studies in healthy volunteers, with limited data in special populations and minimal evaluation of metabolite- or transporter-mediated interactions. This review summarizes the available in vitro and in vivo pharmacokinetic and DDI data for SGLT2 inhibitors, identifies key knowledge gaps related to polypharmacy, metabolite effects, and vulnerable patient groups, and outlines future research priorities to ensure their safe and effective use in real-world clinical practice.

## 1. Introduction

Type 2 diabetes mellitus (T2DM) is a chronic, progressive metabolic disorder marked by insulin resistance and decreasing β-cell function, resulting in sustained hyperglycemia. Its global burden has risen sharply over the past decades: in 2021, an estimated 536.6 million adults (20–79 years) were living with diabetes worldwide, corresponding to a prevalence of 10.5% in this age group. This figure is projected to rise to 12.2% by 2045 [1]. Beyond glycemic dysregulation, T2DM substantially increases the risk of cardiovascular disease (CVD), chronic kidney disease (CKD), and heart failure, which are the primary contributors to morbidity and mortality in this population [2].

Metformin remains the first-line therapy for T2DM and lowers hepatic glucose production primarily through inhibition of mitochondrial complex I and activation of AMP-activated protein kinase. It typically reduces hemoglobin A1c (HbA1c) by 1–2% without causing hypoglycemia or weight gain [3,4,5]. When additional glycemic control is required, other therapeutic classes may be introduced. Sulfonylureas stimulate insulin secretion via pancreatic KATP channel modulation but increase the risk of hypoglycemia and weight gain. Meglitinides act similarly but have shorter binding kinetics, resulting in a lower risk of hypoglycemia [6]. Alpha-glucosidase inhibitors attenuate postprandial glucose excursions by delaying intestinal carbohydrate digestion, resulting in a modest HbA1c reduction of 0.5–0.8%. However, their clinical utility is often limited by their gastrointestinal side effects [7]. Thiazolidinediones activate peroxisome proliferator-activated receptor-γ (PPARγ) to enhance insulin sensitivity and redistribute adipose stores, lowering HbA1c by 0.8–1.5%. However, they frequently cause weight gain and fluid retention and are associated with an increased risk of heart failure [8]. Dipeptidyl peptidase-4 (DPP-4) inhibitors enhance the action of endogenous incretins, leading to a modest HbA1c reduction of 0.5–1.0%, with a neutral effect on body weight and a low risk of hypoglycemia [9]. By contrast, glucagon-like peptide-1 (GLP-1) receptor agonists more robustly activate the incretin pathway, resulting in greater HbA1c reductions of 1.0–1.8%, clinically meaningful weight loss, and cardiovascular risk reduction [10]. However, the requirement for subcutaneous administration and the higher incidence of gastrointestinal adverse events (nausea, vomiting, and diarrhea) limit long-term adherence.

The introduction of sodium-glucose cotransporter 2 (SGLT2) inhibitors has revolutionized the therapeutic landscape for T2DM. SGLT2 mediates secondary active glucose reabsorption by using the sodium gradient generated by the basolateral Na^+^/K^+^-ATPase, enabling glucose transport across the apical membrane against its concentration gradient. By blocking this sodium–glucose cotransport mechanism, SGLT2 inhibitors reduce tubular glucose reabsorption and promote urinary glucose excretion [11]. Since the approval of canagliflozin by the Food and Drug Administration (FDA) of the United States (US) in 2013, other SGLT2 inhibitors—including dapagliflozin, empagliflozin, ertugliflozin, and sotagliflozin—have been introduced into clinical practice. Clinical trials and meta-analyses have consistently demonstrated that SGLT2 inhibitors produce clinically meaningful glycemic reductions when added to background therapy. A recent direct meta-analysis of randomized controlled trials reported a mean placebo-adjusted HbA1c reduction of 0.62% alongside a mean body weight decrease of 0.60 kg after 12 weeks of treatment [12]. Head-to-head comparisons indicate that SGLT2 inhibitors lower HbA1c as effectively as metformin or sulfonylureas, while providing greater weight and blood pressure benefits than DPP-4 inhibitors [13].

Large-scale cardiovascular outcome trials have consistently demonstrated that SGLT2 inhibitors provide benefits beyond glucose control. In the EMPA-REG OUTCOME trial, empagliflozin reduced the risk of 3-point major adverse cardiovascular events (MACE) by 14% (*p* = 0.04) and cardiovascular mortality by 38% (*p* < 0.001) in patients with T2DM and CVD [14]. The CANVAS Program observed that canagliflozin lowered the incidence of the composite endpoint of cardiovascular death, nonfatal myocardial infarction, or nonfatal stroke from 31.5 to 26.9 events per 1000 patient-years (*p* = 0.02), while also reducing hospitalizations for heart failure [15]. In DECLARE-TIMI 58, dapagliflozin significantly decreased the rate of hospitalization for heart failure [16]. Moreover, the DAPA-CKD trial extended dapagliflozin’s indication to CKD, demonstrating relative risk reduction in a composite of sustained ≥50% decline in eGFR, end-stage kidney disease, or renal/cardiovascular death in patients with and without diabetes [17]. Collectively, these pleiotropic effects—including glucose lowering, cardiovascular risk reduction, renoprotection, modest weight loss, blood pressure lowering, and a low risk of hypoglycemia—position SGLT2 inhibitors uniquely for the management of T2DM and its comorbidities. As a reflection of this expanding therapeutic value, major international guidelines currently recommend the use of SGLT2 inhibitors in cardiometabolic disease management. The American Diabetes Association recommends SGLT2 inhibitors for patients with T2DM and CVD or heart failure, independent of baseline HbA1c levels [18]. The European Society of Cardiology recommends SGLT2 inhibitors to reduce heart-failure hospitalizations and improve cardiovascular outcomes [19,20], and the KDIGO guidelines likewise endorse their use as first-line therapy to slow the progression of diabetic kidney disease [21,22]. This convergence of guideline recommendations has led to the widespread integration of SGLT2 inhibitors into routine clinical practice, not only among endocrinologists but also among cardiologists and nephrologists. Consequently, their use has expanded across diverse patient populations, including older adults, those with multiple comorbidities, and individuals at high risk for adverse cardiovascular or renal events.

The therapeutic benefits of SGLT2 inhibitors have driven their widespread use in combination with other glucose-lowering and cardiometabolic therapies, which in turn raises the potential for pharmacokinetic and pharmacodynamic interactions. Dapagliflozin and empagliflozin are now routinely co-prescribed with metformin as first- or second-line therapy, and in more advanced disease, a third agent—either a DPP-4 inhibitor (e.g., sitagliptin) or a GLP-1 receptor agonist (e.g., semaglutide)—is often added, with insulin reserved for refractory hyperglycemia [23]. Furthermore, patients with T2DM frequently receive antihypertensives, lipid-lowering statins, and antithrombotics to mitigate cardiovascular risks. Such therapeutic complexity amplifies the likelihood of clinically relevant drug–drug interactions (DDIs) involving drug-metabolizing enzymes and drug transporters.

Given the growing role of SGLT2 inhibitors across diverse patient populations, a comprehensive and integrated review of their pharmacokinetic profiles and DDI potential is essential. This manuscript summarizes the in vitro and in vivo pharmacokinetics of approved SGLT2 inhibitors, examines their enzyme- and transporter-mediated interaction risks, and highlights remaining knowledge gaps to support safe and effective clinical use.

## 2. Overview of SGLT2 Inhibitors

**Figure 1:**
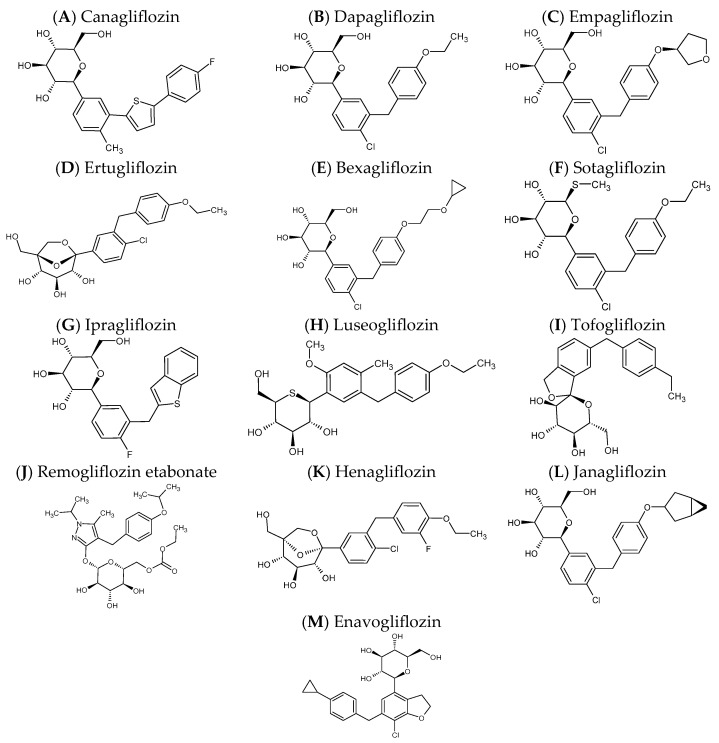
Chemical Structures of Clinically Approved SGLT2 Inhibitors.

Since the first approval of canagliflozin by the US FDA in 2013, the SGLT2 inhibitor class has rapidly expanded to include six agents on the US market, along with several region-specific compounds (Table 1 and Figure 1). Although all SGLT2 inhibitors share a core C-glucoside scaffold that confers high affinity for the renal SGLT2 transporter, structural differences in their aglycone moieties contribute to variations in potency, selectivity, pharmacokinetics, and approved indications.

Canagliflozin (Invokana^®^, 100–300 mg QD; Janssen) was initially introduced for glycemic control in adults and children aged ≥10 years and later received expanded indications for the reduction in cardiovascular events and progression of diabetic nephropathy in high-risk patients [24]. One year later, dapagliflozin (Farxiga^®^, 5–10 mg QD; Bristol-Myers Squibb/AstraZeneca) received similar extensions, with its indications expanding beyond T2DM to include CKD and heart failure, irrespective of diabetic status [25]. Empagliflozin (Jardiance^®^, 10–25 mg QD; Boehringer Ingelheim) received similarly broadened indications for heart failure hospitalization reduction and renal protection in addition to its antihyperglycemic effect [26]. Ertugliflozin (Steglatro^®^, 5–15 mg QD; Merck, 2017) and bexagliflozin (Brenzavvy^®^, 20 mg QD; TheracosBio LLC, 2023) remain focused on glycemic management in adults with T2DM [27,28]. Contrastingly, the latest entrant—the dual SGLT1/SGLT2 inhibitor sotagliflozin (Inpefa^®^, 200–400 mg QD; Lexicon Pharmaceuticals, 2023)—offers additional options for glucose lowering and combined renal and intestinal glucose modulation, respectively [29].

**Table 1:** Information on FDA-approved SGLT2 inhibitors.

Drug	Developed by	Approval Year	Mechanism of Action	Indications	Recommended Dose Regimen	Ref.
Canagliflozin (Invokana^®^)	Janssen	2013	SGLT2 inhibitor	Improves glycemic control in T2DM (adults and pediatric patients aged 10 years and older) Reduces major CV events in T2DM with established CVD Reduces risk of kidney disease progression, CV death, and hospitalization for heart failure in T2DM with diabetic kidney disease	100–300 mg QD	[24]
Dapagliflozin (Farxiga^®^)	Bristol-Myers Squibb/ Astrazeneca	2014	SGLT2 inhibitor	Reduces risks of kidney disease progression, CV death, and hospitalization for heart failure in CKD Reduces CV death and heart failure events in heart failure Reduces hospitalization for heart failure risk in T2DM with CVD or risk factors Improves glycemic control in T2DM (adults and pediatric patients aged 10 years and older)	5–10 mg QD	[25]
Empagliflozin (Jardiance^®^)	Boehringer Ingelheim	2014	SGLT2 inhibitor	Reduces risk of CV death and hospitalization in heart failure. Reduces risk of kidney disease progression and CV events in CKD Reduces risk of CV death in T2DM with established CV disease. Improves glycemic control in T2DM (adults and pediatric patients aged 10 years and older)	10–25 mg QD	[26]
Ertugliflozin (Steglatro^®^)	Merk	2017	SGLT2 inhibitor	Improves glycemic control in T2DM (adults)	5–15 mg QD	[27]
Bexagliflozin (Brenzavvy^®^)	TheracosBio LLC	2023	SGLT2 inhibitor	Improves glycemic control in T2DM (adults)	20 mg QD	[28]
Sotagliflozin (Inpefa^®^)	Lexicon Pharmaceuticals	2023	SGLT1/2 inhibitor	Reduces CV death and heart failure events in adults with heart failure or with T2DM, CKD, and CV risk factors	200–400 mg QD	[29]

CKD, chronic kidney disease; CV, cardiovascular; CVD, cardiovascular disease; SGLT2, sodium glucose cotransporter 2; T2DM, type 2 diabetic mellitus; QD, once daily.

Beyond the FDA-approved inhibitors, several regionally developed SGLT2 inhibitors have been approved and are currently in clinical use, particularly in Asia. These include ipragliflozin, luseogliflozin, and tofogliflozin, all approved in Japan in 2014 [30,31,32], as well as remogliflozin etabonate (India), henagliflozin and janagliflozin (China), and enavogliflozin (South Korea) [33,34,35]. Although these agents share similar mechanisms, their approval pathways, clinical data availability, and market penetration vary by region.

In terms of safety, SGLT2 inhibitors are generally well tolerated, with a low intrinsic risk of hypoglycemia when used as monotherapy because of their insulin-independent mechanism of action [36]. Nonetheless, class-related adverse events are well-documented and merit careful attention. Genital mycotic infections, particularly in women, are among the most frequently reported side effects, occurring in up to 10% of patients, and are largely due to glucosuria-induced microbial overgrowth in the genitourinary tract [37]. Other common adverse effects include volume depletion and hypotension, particularly in older adult patients or those receiving concomitant diuretics, owing to the osmotic diuretic effect of SGLT2 inhibition [38]. Urinary tract infections have also been reported, although their causality remains less clear than that of genital infections [39]. More serious, although rare, adverse events include diabetic ketoacidosis (DKA), which often presents as a euglycemic state. This atypical presentation, termed “euglycemic DKA”, may delay diagnosis and is thought to arise from increased glucagon levels, enhanced lipolysis, and ketogenesis under conditions of insulinopenia or stress. The US FDA and European Medicines Agency have issued warnings regarding this risk, particularly in patients with low insulin reserves, alcohol use, or during perioperative fasting [40,41].

Given the abovementioned established safety concerns associated with SGLT2 inhibitors, as well as their increasing use in combination with other medications for glycemic control, cardiovascular protection, and renal preservation, a comprehensive understanding of their pharmacokinetic characteristics and DDI potential is essential.

## 3. In Vitro Pharmacokinetics of SGLT2 Inhibitors

The in vitro pharmacokinetic characterization of SGLT2 inhibitors provides foundational insights into their metabolic pathways, distribution, and potential for pharmacokinetic DDIs. Although SGLT2 inhibitors share a common mechanism of inhibiting renal glucose reabsorption, they differ in their metabolic stability, enzyme affinity, and transporter liabilities, all of which influence their clinical pharmacology (Table 2).

SGLT2 inhibitors differ in their solubility and permeability profiles, and are classified as BCS Class I to IV. Despite the low aqueous solubility of some agents, adequate exposure is typically achievable because of their effective permeability and low-dose requirements. SGLT2 inhibitors exhibit high plasma protein binding (generally > 80%), except for remogliflozin, an active metabolite of remogliflozin etabonate (65% bound to plasma protein) [51].

**Figure 2:**
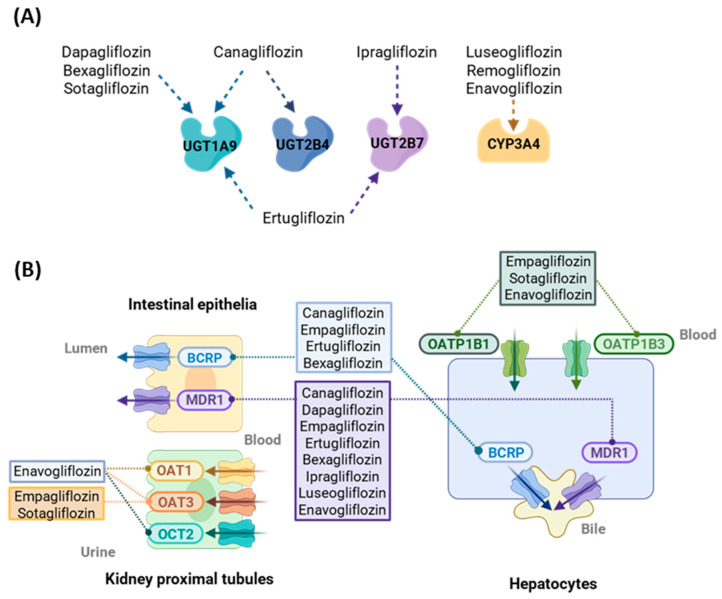
Involvement of metabolizing enzymes and transporters in the pharmacokinetics of SGLT2 inhibitors. (**A**) Drug metabolizing enzymes responsible for the elimination of SGLT2 inhibitors. (**B**) Drug transporters that transport SGLT2 inhibitors across the membrane in the intestine, liver, and kidney.

A predominant feature of many SGLT2 inhibitors is their reliance on phase II glucuronidation pathways for metabolism (Figure 2A). The most common uridine diphosphate glucuronosyltransferases (UGTs) are UGT1A9, UGT2B4, and UGT2B7. For example, dapagliflozin is predominantly metabolized by UGT1A9 to form an inactive 3-O-glucuronide (M15), whereas canagliflozin is primarily conjugated to UGT1A9 and UGT2B4 to form M7 and M5 metabolites [44]. Ertugliflozin and empagliflozin follow similar metabolic routes involving UGT1A9 and UGT2B7, although the contribution of metabolism to their overall in vivo clearance differs [45,46]. Unlike most SGLT2 inhibitors, which rely primarily on UGT-mediated metabolism, luseogliflozin undergoes substantial CYP3A4/5 metabolism [50], warranting closer consideration for CYP-mediated drug interactions.

In vitro enzyme inhibition studies have demonstrated that most SGLT2 inhibitors do not significantly inhibit major CYP isoforms at clinically relevant concentrations. Canagliflozin shows weak inhibitory activity against CYP3A4 [half maximal inhibitory concentration (IC_50_) = 27 µM], CYP2C8 (IC_50_ = 75 µM), and CYP2B6 (IC_50_ = 16 µM), but given its lower therapeutic plasma concentrations and high protein binding, the clinical relevance of this inhibition is expected to be limited [42]. Other inhibitors such as dapagliflozin, empagliflozin, and ertugliflozin show negligible inhibition across major CYP enzymes (including CYP1A2, CYP2C9, CYP2D6, and CYP3A4) with IC_50_ values typically exceeding 40 µM [44,45,46]. Regarding UGT enzyme inhibition, most SGLT2 inhibitors show weak or no inhibitory activity. Canagliflozin exhibits modest inhibition toward UGT1A1 and UGT1A6 (IC_50_ = 91 and 50 µM, respectively), whereas dapagliflozin shows weak UGT1A9 and UGT1A10 inhibition in the range of 39–66 µM. However, these values exceed the concentrations achieved in vivo; therefore, the potential for UGT-mediated DDIs is low.

The enzyme induction potential of these agents has also been evaluated in vitro using human hepatocyte assays. The commonly used SGLT2 inhibitors—including canagliflozin, dapagliflozin, empagliflozin, or ertugliflozin—showed no significant induction of CYP1A2, CYP2B6, or CYP3A4 at concentrations in the range of 20–30 µM. One exception is the glucuronide metabolite M19 of sotagliflozin, which has been shown to induce CYP3A4 mRNA expression, suggesting the potential for metabolite-mediated DDIs [48].

Most SGLT2 inhibitors are substrates of efflux transporters such as MDR1 and BCRP, and some also interact with MRP2 (Figure 2B). In contrast, interactions with uptake transporters including organic anion transporter 1/3 (OAT1/3), organic anion transporting polypeptide 1B1/1B3 (OATP1B1/1B3), and organic cation transporter 1/2 (OCT1/2) are generally limited. Some agents, such as empagliflozin and sotagliflozin, are weak substrates of OAT3 and OATP1B1/1B3; however, these interactions appear to be minor under in vitro conditions [45,48]. The transporter inhibition potential of these agents is also low, with most compounds exhibiting IC_50_ values far above the experimentally relevant concentrations. Exceptions are canagliflozin, which weakly inhibits MDR1 and MRP2, and bexagliflozin, which moderately inhibits OATP1B1 and multidrug and toxin extrusion protein (MATE) 1 in vitro at high concentrations [43,47].

## 4. Clinical Pharmacokinetics of SGLT2 Inhibitors

**Figure 3:**
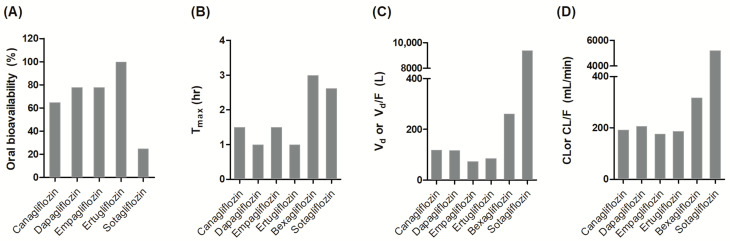
Comparison of pharmacokinetic properties of SGLT2 inhibitors. Oral bioavailability (**A**), T_max_ (**B**), V_d_ (**C**) (presented as V_d_/F for empagliflozin, bexagliflozin, and sotagliflozin), and CL (**D**) (presented as CL/F for empagliflozin, bexagliflozin, and sotagliflozin) are shown for SGLT2 inhibitors.

SGLT2 inhibitors exhibit broadly similar pharmacokinetic characteristics, which support once-daily oral dosing across the class. However, distinct inter-drug differences exist in terms of oral bioavailability, food effects, elimination pathways, and sensitivity to organ dysfunction, which are important considerations for individualized treatment (Table 3 and Figure 3).

### 4.1. Absorption and Food Effects

Most SGLT2 inhibitors demonstrate moderate-to-high oral bioavailability, typically ranging from 65% (canagliflozin) to 100% (ertugliflozin) [43,44,45,46,47]. One exception is sotagliflozin, which has a reported bioavailability of only 25% [48]. The time to reach maximum concentration (T_max_) values for SGLT2 inhibitors ranges from 1 to 4 h post administration, with the slowest absorption observed for bexagliflozin (2–4 h) [47]. Although food can alter absorption kinetics across the class, only sotagliflozin demonstrates a clinically meaningful food effect. Most agents (e.g., canagliflozin, empagliflozin, dapagliflozin, ertugliflozin, and bexagliflozin) show modest changes in maximum drug concentration (C_max_) or T_max_ without significantly affecting the area under the curve (AUC), with these changes not considered clinically meaningful. Conversely, sotagliflozin demonstrates pronounced food effects, with fed conditions leading to a 149% increase in C_max_ and a 50% increase in AUC compared with fasting conditions [48]. Clinical studies have further demonstrated that administering 400 mg of sotagliflozin immediately before breakfast, 30 min before, or 1 h before produces consistent pharmacodynamic effects on urine glucose excretion, insulin, and postprandial glucose levels. Therefore, sotagliflozin should be taken within 1 h before the first meal of the day.

### 4.2. Distribution and Elimination Profiles

SGLT2 inhibitors display a broad range of volume of distribution (V_d_), reflecting differences in tissue penetration and physicochemical properties. Most agents, such as dapagliflozin, ertugliflozin, canagliflozin, and empagliflozin exhibit moderate V_d_ values between 70 and 120 L, suggesting a distribution beyond the plasma compartment but without extensive tissue accumulation [43,44,45,46]. By contrast, bexagliflozin (262 L) and sotagliflozin (9392 L) have substantially higher V_d_ values, indicating extensive tissue affinity or non-specific binding [47,48].

Although SGLT2 inhibitors undergo both renal and fecal elimination, the proportion and chemical form excreted vary across agents. Empagliflozin is primarily excreted as an unchanged parent compound, indicating minimal metabolic transformation (<10%) [45]. By contrast, dapagliflozin undergoes extensive glucuronidation, with less than 2% of the dose excreted unchanged in the urine, and over 70% eliminated as a metabolite [44]. Approximately 21% of the dapagliflozin dose is recovered in feces, of which approximately 15% is attributed to the unchanged drug, suggesting partial biliary excretion or elimination of the unabsorbed drug. Canagliflozin and bexagliflozin are predominantly excreted in urine as glucuronide conjugates, with only minimal amounts of the parent drug recovered. However, their fecal elimination profiles differ; approximately 80% of fecally excreted canagliflozin remains unchanged, whereas for bexagliflozin, approximately half of the fecal recovery represents the parent compound [43,47]. Ertugliflozin exhibits a mixed elimination profile with roughly equal recoveries in urine and feces. Although only 1.5% of the ertugliflozin dose is excreted unchanged in the urine, nearly 80% of the fecal recovery is unchanged drug, similar to that of canagliflozin [46]. Sotagliflozin is cleared via both the renal (57%) and fecal (37%) routes, with excretion occurring as both unchanged drug and glucuronide conjugates [48].

Across the class, the terminal elimination half-lives range from approximately 10 to 17 h, supporting once-daily dosing. Furthermore, all SGLT2 inhibitors demonstrate dose-proportional pharmacokinetics within their therapeutic ranges, enabling predictable systemic exposure and flexible titration in clinical settings.

### 4.3. Renal and Hepatic Impairment

The pharmacokinetic profiles of SGLT2 inhibitors are variably influenced by renal and hepatic impairment, with the degree of change largely dependent on the metabolic and elimination pathways of each drug as shown in Table 3. As renal glucose reabsorption is the primary therapeutic target of SGLT2 inhibitors, renal function not only affects drug exposure but may also influence the pharmacodynamic response.

In patients with renal impairment, systemic exposure to SGLT2 inhibitors tends to increase to varying extents. Dapagliflozin shows a marked increase in exposure, with the AUC rising by approximately 45%, 100%, and 200% in patients with mild, moderate, and severe renal impairment, respectively [44]. Similarly, sotagliflozin shows pronounced increases in AUC, reaching 70% and 170% in mild and moderate impairment, respectively. Ertugliflozin and empagliflozin exhibit moderate increases in AUC (60–70% and up to 66%, respectively) across impairment levels [48]. By contrast, canagliflozin and bexagliflozin demonstrate smaller increases, with AUC elevations of 15–53% and 7–54%, respectively, suggesting relatively less dependence on renal function for overall clearance [43,47]. These differences likely reflect the extent of renal clearance and the metabolism of each agent via glucuronidation.

Hepatic impairment also affects the pharmacokinetics of SGLT2 inhibitors to varying extents. Dapagliflozin and empagliflozin exhibit moderate increases in exposure under hepatic impairment, with C_max_ and AUC values increasing by up to 40% and 67%, respectively, for dapagliflozin, and by 48% and 75%, respectively, for empagliflozin in cases of severe impairment [44,45]. Canagliflozin and bexagliflozin show smaller changes in exposure in mild to moderate hepatic impairment (≤28% increase in AUC), whereas ertugliflozin shows relatively modest effects (approximately 21% increase in C_max_) [43,47]. Interestingly, sotagliflozin does not exhibit a significant increase in AUC in mild hepatic impairment, although data on moderate-to-severe impairment suggest a more substantial effect (3–6-fold increase in AUC) [48].

Overall, while all SGLT2 inhibitors are affected to some extent by renal and hepatic dysfunction, dapagliflozin and sotagliflozin appear to be more sensitive to renal impairment, whereas empagliflozin shows notable changes in both renal and hepatic settings.

### 4.4. Disease Conditions

It is well established that cardiovascular diseases such as heart failure can influence the pharmacokinetics of various drugs due to altered gastrointestinal perfusion, hepatic and renal blood flow, changes in plasma protein binding, and expanded extracellular fluid volume [57,58,59]. These pathophysiological changes often result in reduced drug absorption, delayed distribution, and impaired clearance in patients with heart failure [59].

There is emerging evidence that heart failure may influence the pharmacokinetics of certain SGLT2 inhibitors. For instance, a pooled analysis demonstrated that the systemic exposure to dapagliflozin in patients with heart failure with reduced ejection fraction (HFrEF) was approximately 1.2-fold higher than in patients with T2DM, a difference considered not clinically significant [60]. Similarly, Rascher et al. (2024) reported that the steady-state trough concentrations of empagliflozin 10 mg in patients with heart failure (with or without T2DM) were 1.47–1.53-fold higher than in T2DM patients receiving the same dose, yet still below the exposure achieved with the 25 mg dose [61].

These findings suggest that while heart failure may lead to modest increases in systemic exposure of certain SGLT2 inhibitors, these changes remain within a therapeutically acceptable range and do not necessitate dose adjustment for dapagliflozin or empagliflozin. Nevertheless, given the absence of comparable pharmacokinetic data for other SGLT2 inhibitors such as canagliflozin and ertugliflozin, further studies are warranted to assess whether this observation can be generalized across the class.

### 4.5. Impact of Demographics

Demographic factors, including age, body weight, sex, and race, do not have a clinically significant effect on the pharmacokinetics of any agent in the class. This conclusion is based primarily on population pharmacokinetic (PopPK) analyses conducted across multiple clinical studies rather than dedicated demographic subgroup trials [43,44,45,46]. Although individual PK studies rarely reported detailed demographic characteristics, integrated PopPK analyses consistently support fixed dosing without the need for demographic-based adjustments.

## 5. Pre-Clinical Drug Interactions of SGLT2 Inhibitors

Pre-clinical studies in rodent models provide mechanistic insights into potential pharmacokinetic interactions between SGLT2 inhibitors and compounds that modulate metabolic enzymes or transporters (Table 4). While the extrapolation of these findings to humans must be approached cautiously, these data highlight the importance of considering both metabolic and transporter-mediated mechanisms in potential DDIs involving this drug class.

### 5.1. Metabolism-Based Interactions

Both canagliflozin and dapagliflozin, which are extensively glucuronidated by UGT1A9, exhibit increased systemic exposure in rats when co-administered with donafenib, a known substrate and inhibitor of UGT1A9 [62]. These interactions are bidirectional; canagliflozin increases the C_max_ of donafenib by 1.77-fold, whereas donafenib increases the AUC of canagliflozin by 1.29-fold. These effects suggest competitive inhibition at the binding site of Ugt1a7 (rat homolog of human UGT1A9), with implications for the altered clearance of either drug.

Among SGLT2 inhibitors, ertugliflozin shows the highest sensitivity to enzyme-mediated interactions, likely because of its significant dependence on both UGT1A9 and UGT2B7 for elimination. Co-administration with ketoconazole, a known inhibitor of CYP3A4 and certain UGT isoforms, was shown to result in a 3.3-fold increase in AUC and a 70% reduction in clearance in rats [63]. Because CYP3A4 plays only a minor role in ertugliflozin metabolism, the observed exposure changes are more likely attributable to UGT inhibition [63]. These findings reinforce the central role of UGT-mediated clearance in the metabolic disposition of ertugliflozin.

### 5.2. Transporter-Mediated DDIs

Canagliflozin, a weak inhibitor of MDR1 and MRP2, did not significantly alter sorafenib pharmacokinetics in rats (C_max_ 1.15-fold, AUC 1.03-fold). This likely reflects insufficient inhibitory potency at the tested dose or saturation of sorafenib’s transporter-mediated efflux [64]. However, the co-administration of lenvatinib, another MDR1 and MRP2 substrate, with canagliflozin led to modest increases in exposure to lenvatinib (C_max_ and AUC increased by 1.37- and 1.29-fold, respectively) [64]. Moreover, canagliflozin exhibited increased exposure when administered with sorafenib (C_max_ 1.33-fold, AUC_inf_ 1.38-fold), suggesting that sorafenib may inhibit canagliflozin efflux via shared transporters such as MDR1 or BCRP.

**Table 4:** Pre-clinical drug interaction studies of SGLT2 inhibitors.

SGLT2 Inhibitor	Perpetrator		Victim		Species/Condition	Ratio (C_max_, AUC) ^a^	Ref.
	Drug	Dose Regimen	Drug	Dose Regimen			
Canagliflozin	Canagliflozin	10 mg/kg, PO	Sorafenib	100 mg/kg, PO	Normal rat	1.15, 1.03	[64]
	Canagliflozin	10 mg/kg, PO	Lenvatinib	1.2 mg/kg, PO	Normal rat	1.37, 1.29	[64]
	Canagliflozin	10 mg/kg, PO	Donafenib	40 mg/kg, PO for 7 days	Normal rat	1.77, 1.37	[62]
	Sorafenib	100 mg/kg, PO	Canagliflozin	10 mg/kg, PO	Normal rat	1.33, 1.38	[64]
	Lenvatinib	1.2 mg/kg, PO	Canagliflozin	10 mg/kg, PO	Normal rat	0.97, 1.40	[64]
	Donafenib	40 mg/kg, PO for 7 days	Canagliflozin	10 mg/kg, PO	Normal rat	0.87, 1.29	[62]
	Myricetin	6 mg/kg, PO	Canagliflozin	10 mg/kg PO	Normal rat	1.25, 1.14	[65]
	Myricetin	6 mg/kg, PO for 8 days	Canagliflozin	10 mg/kg PO	Normal rat	1.40, 1.19	[65]
	Myricetin	6 mg/kg, PO	Canagliflozin	10 mg/kg PO	Dietetic rat	1.26, 1.13	[65]
	Myricetin	6 mg/kg, PO for 8 days	Canagliflozin	10 mg/kg PO	Dietetic rat	1.39, 1.15	[65]
Dapagliflozin	Dapagliflozin	1 mg/kg	Donafenib	40 mg/kg, PO for 7 days	Normal rat	1.37, 0.97	[62]
	Dapagliflozin	1 mg/kg PO for 7 days	Sorafenib	100 mg/kg PO for 7 days	Normal rat	0.58, 0.54	[66]
	Dapagliflozin	0.5 mg/kg, PO	Sorafenib	100 mg/kg, PO	Normal rat	1.26, 1.36	[67]
	Dapagliflozin	1 mg/kg, PO	Sorafenib	100 mg/kg, PO	Normal rat	1.53, 1.38	[67]
	Donafenib	40 mg/kg, PO for 7 days	Dapagliflozin	1 mg/kg	Normal rat	0.85, 1.77	[62]
	Sorafenib	100 mg/kg, PO for 7 days	Dapagliflozin	1 mg/kg, PO for 7 days	Normal rat	1.03, 1.80	[66]
	LCZ696	40 mg/kg, PO	Dapagliflozin	2 mg/kg, PO	Normal rat	1.30, 1.27	[68]
	LCZ696	40 mg/kg, IV	Dapagliflozin	2 mg/kg, IV	Normal rat	1.12 (AUC ratio)	[68]
	Sorafenib	100 mg/kg, PO	Dapagliflozin	0.5 mg/kg, PO	Normal rat	1.07, 1.11	[67]
	Sorafenib	100 mg/kg, PO	Dapagliflozin	1 mg/kg, PO	Normal rat	1.00, 1.10	[67]
Empagliflozin	Empagliflozin	1.5 mg/kg, PO	Fluvastatin	2 mg/kg, PO	Normal rabbit	1.41, 2.12	[69]
	Acai berry	250 mg/day, PO for 10 days	Empagliflozin	2.5 mg/kg, PO	Normal rat	1.44, 1.32	[70]
	Grapefruit juice	10 mL/day, PO for 4 days	Empagliflozin	0.16 mg/kg, PO	Normal rat	2.61, 1.11	[71]
Ertugliflozin	Mefenamic acid	20 mg/kg, IV	Ertugliflozin	0.5 mg/kg, IV	Normal rat	1.38 (AUC ratio)	[63]
	Mefenamic acid	20 mg/kg, PO	Ertugliflozin	0.5 mg/kg, PO	Normal rat	1.01, 1.19	[63]
	Ketoconazole	20 mg/kg, IV	Ertugliflozin	0.5 mg/kg, IV	Normal rat	3.32 (AUC ratio)	[63]
	Ketoconazole	20 mg/kg, PO	Ertugliflozin	0.5 mg/kg, PO	Normal rat	1.48, 2.95	[63]
	Sinapic acid	20 mg/kg, PO	Ertugliflozin	20 mg/kg, PO	Normal rat	1.26, 1.15	[72]
	Sinapic acid	20 mg/kg, PO for 7 days	Ertugliflozin	20 mg/kg, PO	Normal rat	2.19, 1.51	[72]
	Sinapic acid	20 mg/kg, PO	Ertugliflozin	20 mg/kg, PO	Diabetic rat	1.42, 1.34	[72]
	Sinapic acid	20 mg/kg, PO for 7 days	Ertugliflozin	20 mg/kg, PO	Diabetic rat	2.43, 1.82	[72]
Luseogliflozin	Luseogliflozin	0.1 mg/kg, PO	Miglitol	1.5 mg/kg, PO	Normal rat	0.97, 1.12 ^b^	[73]

^a^ Calculated by dividing C_max_ or AUC of victim in the absence of perpetrator by the one in the presence of perpetrator. ^b^ Presented as geometric mean ratio.

Empagliflozin displays transporter-mediated interactions through distinct mechanisms. Co-administration with fluvastatin led to a significant increase in fluvastatin AUC (2.1-fold) in normal rabbits, potentially via the inhibition of hepatic OATP by empagliflozin [69]. In addition, grapefruit juice markedly increased empagliflozin exposure (C_max_ 2.6-fold) [71]. Although grapefruit juice is a known inhibitor of intestinal CYP3A, this is unlikely to be the primary mechanism as empagliflozin undergoes minimal CYP-mediated metabolism. The observed increase is more likely attributable to the inhibition of intestinal MDR1.

## 6. Clinical Drug Interactions of SGLT2 Inhibitors with Antidiabetic Agents

### 6.1. Interactions of SGLT2 Inhibitors with Metformin

Accumulating clinical evidence indicates that SGLT2 inhibitors show minimal clinically relevant pharmacokinetic interactions with metformin (Table 5). Across a range of studies involving healthy volunteers and patients with T2DM, co-administration of metformin with various SGLT2 inhibitors—including canagliflozin, dapagliflozin, ertugliflozin, ipragliflozin, luseogliflozin—resulted in only modest changes in metformin exposure [74,75,76,77,78,79,80,81,82]. Most changes in C_max_ and AUC values were within the acceptable bioequivalence range (80–125%), and no dose adjustments were required in clinical practice. Slight increases in metformin exposure observed with canagliflozin (AUC 1.2-fold) are likely mediated by weak inhibition of transporters such as OCT1 and OCT2 (IC_50_ = 5.2 and 44 μM, respectively) [74]. OCT2 is involved in the active tubular secretion of metformin, which is the primary route of its elimination. However, the magnitude of the interaction is limited, with an increase in metformin AUC of 20%, and has not been associated with safety concerns such as accumulation-related adverse events.

Metformin does not significantly alter the pharmacokinetics of SGLT2 inhibitors because it is not a known modulator of CYP450 or UGT enzymes, nor does it interact with hepatic uptake transporters involved in their metabolism or distribution. In studies where metformin was the perpetrator drug, exposure to SGLT2 inhibitors remained unchanged or fluctuated within a narrow range, which was not considered pharmacologically meaningful as presented in Table 5.

Taken together, these findings underscore the favorable interaction profiles of SGLT2 inhibitors in combination with metformin. From both pharmacokinetic and clinical perspectives, the co-administration of these agents is considered safe, predictable, and unlikely to require therapeutic monitoring or dose adjustments in routine clinical settings.

### 6.2. Interactions of SGLT2 Inhibitors with DPP4-Inhibitors

SGLT2 and DPP-4 inhibitor combination therapy is widely used for the treatment of T2DM, with numerous studies having evaluated their potential pharmacokinetic interactions (Table 6). Across all tested combinations, including dapagliflozin with sitagliptin, empagliflozin with linagliptin, and ertugliflozin with saxagliptin, no meaningful changes in C_max_ or AUC were observed. Most studies reported that exposure ratios (combination vs. monotherapy) remained within the standard bioequivalence range (0.80–1.25), suggesting minimal interaction [75,76,77,79,80,83,84,85,86,87,88]. For example, the co-administration of ertugliflozin and sitagliptin met all bioequivalence criteria, whereas empagliflozin combined with linagliptin or sitagliptin produced only modest C_max_ changes (10–12%), which did not warrant dose adjustment [76,88]. Similarly, administration of canagliflozin with teneligliptin and dapagliflozin with saxagliptin or evogliptin showed negligible changes systemically [84,85]. Mechanistically, this lack of interaction can be explained by the distinct metabolic and elimination pathways of the two drug classes. SGLT2 inhibitors are primarily cleared by glucuronidation or renal excretion, whereas DPP-4 inhibitors are eliminated via renal, biliary, or CYP-mediated routes, with no significant overlap.

**Table 5:** Clinical drug interaction studies of SGLT2 inhibitors with metformin.

Perpetrator		Victim		Subjects	GMR [C_max_, AUC (90% CI)] ^a^	Ref.
Drug	Dosing Regimen	Drug	Dosing Regimen			
SGLT2 inhibitors as perpetrators						
Canagliflozin	300 mg/day, MD	Metformin	2000 mg (IR)	HV	1.06 (0.93–1.20), 1.20 (1.08–1.34)	[74]
Dapagliflozin	50 mg	Metformin	1000 mg	HV	0.95 (0.87–1.05), 1.00 (0.93–1.08)	[75]
Ertugliflozin	15 mg	Metformin	1000 mg	HV	0.93, 0.96 ^b^	[76]
Luseogliflozin	5 mg	Metformin	250 mg	HV	1.00 (0.90–1.11), 1.04 (0.95–1.14)	[77]
Ipragliflozin	300 mg	Metformin	800–1500 mg BID, MD	T2DM	1.11 (1.03–1.19), 1.18 (1.08–1.28)	[78]
Tofogliflozin	40 mg	Metformin	750 mg	HV	1.09 (1.00–1.19), 1.08 (1.01–1.16)	[79]
Enavogliflozin	2 mg	Metformin	1000 mg TID, MD	HV	0.98 (0.90–1.06), 1.05 (0.98–1.13)	[89]
Enavogliflozin	2 mg	Gemigliptin/ Metformin	50 mg/day + 1000 mg (IR) TID, MD	HV	1.05 (0.99–1.12), 1.03 (0.98–1.09)	[80]
Henagliflozin	25 mg/day, MD	Metformin	1000 mg	HV	1.12 (1.02–1.23), 1.09 (1.02–1.16)	[82]
SGLT2 inhibitors as victims						
Metformin	2000 mg (IR)	Canagliflozin	300 mg/day, MD	HV	1.05 (0.96–1.16), 1.10 (1.05–1.15)	[74]
Metformin	1000 mg	Dapagliflozin	20 mg	HV	0.93 (0.85–1.02), 1.00 (0.94–1.05)	[75]
Metformin	1000 mg	Ertugliflozin	15 mg	HV	0.96, 1.02 ^b^	[76]
Metformin	250 mg	Luseogliflozin	5 mg	HV	0.93 (0.85–1.01), 1.00 (0.97–1.02)	[77]
Metformin	750 mg	Tofogliflozin	40 mg	HV	1.08 (0.97–1.20), 1.02 (0.98–1.07)	[79]
Metformin	1000 mg TID, MD	Enavogliflozin	2 mg	HV	1.22 (1.13–1.31), 1.09 (1.05–1.14)	[89]
Gemigliptin/ Metformin	50 mg/day + 1000 mg (IR) TID, MD	Enavogliflozin	2 mg	HV	1.27 (1.20–1.35), 1.17 (1.12–1.22)	[80]
Metformin	1000 mg	Henagliflozin	25 mg/day, MD	HV	0.99 (0.92–1.07), 1.08 (1.04–1.12)	[82]

BID, twice daily; CI, confidence interval; HV, healthy volunteers; IR, immediate release; MD, multiple dosing; T2DM, type 2 diabetes mellitus; TID, three times daily. ^a^ Geometric mean ratio of C_max_ or AUC of the victim drug in the presence of the perpetrator to that in its absence. ^b^ Presented as arithmetic mean ratio.

**Table 6:** Clinical drug interaction studies of SGLT2 inhibitors with DPP4 inhibitors.

Perpetrator		Victim		Subjects	GMR [C_max_, AUC (90% CI)] ^a^	Ref.
Drug	Dosing Regimen	Drug	Dosing Regimen			
SGLT2 inhibitors as perpetrators						
Canagliflozin	200 mg/day, MD	Teneligliptin	40 mg	HV	0.98 (0.90–1.06), 0.98 (0.94–1.03)	[84]
Dapagliflozin	20 mg	Sitagliptin	100 mg	HV	0.89 (0.81–0.97), 1.01 (0.99–1.04)	[75]
Dapagliflozin	10 mg	Saxagliptin	5 mg	HV	0.93 (0.88–0.97), 0.99 (0.96–1.02) [5-OH saxagliptin] 1.06 (1.00–1.11), 1.09 (1.06–1.11)	[85]
Dapagliflozin	10 mg/day, MD	Evogliptin	5 mg/day, MD	HV	1.03 (0.96–1.11), 1.00 (0.95–1.06)	[86]
Empagliflozin	25 mg/day, MD	Evogliptin	5 mg/day, MD	HV	1.01 (0.89–1.15), 1.00 (0.88–1.14)	[86]
Empagliflozin	50 mg/day, MD	Sitagliptin	100 mg/day, MD	HV	1.09 (1.01–1.17), 1.03 (0.99–1.07)	[87]
Empagliflozin	50 mg/day, MD	Linagliptin	5 mg/day, MD	HV	1.01 (0.87–1.19), 1.03 (0.96–1.11)	[88]
Ertugliflozin	15 mg	Sitagliptin	100 mg	HV	1.01, 1.02 ^b^	[76]
Luseogliflozin	5 mg	Sitagliptin	50 mg	HV	0.98 (0.92–1.05), 1.03 (1.01–1.05)	[77]
Ipragliflozin	150 mg, MD	Sitagliptin	100 mg	HV	0.92 (0.83–1.03), 1.00 (0.97–1.04)	[83]
Tofogliflozin	40 mg	Sitagliptin	100 mg	HV	0.88 (0.78–0.98), 1.03 (1.00–1.05)	[79]
Enavogliflozin	2 mg	Gemigliptin/ Metformin	50 mg/day + 1000 mg (IR) TID, MD	HV	[Gemigliptin] 1.05 (0.98–1.12), 1.04 (1.02–1.06)	[80]
SGLT2 inhibitors as victims						
Teneligliptin	40 mg/day, MD	Canagliflozin	200 mg	HV	0.98 (0.88–1.10), 0.98 (0.96–1.01)	[84]
Sitagliptin	100 mg	Dapagliflozin	20 mg	HV	0.96 (0.88–1.05), 1.08 (1.03–1.13)	[75]
Saxagliptin	5 mg	Dapagliflozin	10 mg	HV	0.94 (0.87–1.02), 0.99 (0.97–1.01)	[85]
Evogliptin	5 mg/day, MD	Dapagliflozin	10 mg/day, MD	HV	1.09 (0.95–1.25), 1.02 (0.99–1.05)	[86]
Evogliptin	5 mg/day, MD	Empagliflozin	25 mg/day, MD	HV	0.99 (0.88–1.12), 1.04 (1.00–1.08)	[86]
Linagliptin	5 mg/day, MD	Empagliflozin	50 mg/day, MD	HV	0.88 (0.79–0.99), 1.02 (0.97–1.07)	[88]
Sitagliptin	100 mg/day, MD	Empagliflozin	50 mg/day, MD	HV	1.08 (0.97–1.19), 1.10 (1.04–1.17)	[87]
Sitagliptin	100 mg	Ertugliflozin	15 mg	HV	0.98, 1.02 ^b^	[76]
Sitagliptin	50 mg	Luseogliflozin	5 mg	HV	0.97 (0.91–1.02), 0.99 (0.97–1.02)	[77]
Sitagliptin	100 mg/day, MD	Ipragliflozin	150 mg	HV	0.97 (0.90–1.03), 0.95 (0.93–0.97)	[83]
Sitagliptin	100 mg	Tofogliflozin	40 mg	HV	0.96 (0.86–1.06), 1.02 (1.00–1.05)	[79]
Gemigliptin/ Metformin	50 mg/day + 1000 mg (IR) TID, MD	Enavogliflozin	2 mg	HV	1.27 (1.20–1.35), 1.17 (1.12–1.22)	[80]

HV, healthy volunteers; IR, intermediate release; MD, multiple dosing; TID, three times daily. ^a^ Geometric mean ratio of C_max_ or AUC of the victim drug in the presence of the perpetrator to that in its absence. ^b^ Presented as arithmetic mean ratio.

Taken together, SGLT2 and DPP-4 inhibitors can be co-administered without dose adjustment because their pharmacokinetics are largely independent. This favorable interaction profile supports their widespread use as dual therapy in clinical practice.

### 6.3. Interactions of SGLT2 Inhibitors with Thiazolidinedione Antidiabetic Drugs

SGLT2 inhibitors and thiazolidinediones are frequently co-administered to manage T2DM because of their complementary mechanisms of action. SGLT2 inhibitors lower blood glucose by promoting glucosuria through inhibition of renal glucose reabsorption, whereas thiazolidinediones enhance insulin sensitivity by activating peroxisome PPARγ in adipose and muscle tissues. SGLT2 inhibitors, such as dapagliflozin, empagliflozin, ipragliflozin, and tofogliflozin, are primarily metabolized through glucuronidation mediated by UGTs, including UGT1A9 and UGT2B7. Contrastingly, thiazolidinediones such as pioglitazone and lobeglitazone undergo hepatic metabolism mainly via CYP450, including CYP2C8, CYP3A4, and CYP2C9. This distinct separation of the metabolic pathways reduces their potential for metabolic competition or inhibition. However, considering the clinical relevance of polypharmacy in diabetes management, several studies have evaluated the potential DDIs between these two classes (Table 7).

Numerous pharmacokinetic studies in healthy volunteers confirmed the absence of clinically significant interactions between SGLT2 inhibitors and thiazolidinediones. For example, co-administration of dapagliflozin (50 mg) and pioglitazone (45 mg) showed no significant change in pioglitazone exposure, with C_max_ and AUC ratios close to 1 [75]. Similarly, empagliflozin administered at therapeutic and supratherapeutic doses (10–50 mg) with pioglitazone resulted in minimal alterations in the pharmacokinetics of either the drug or its active metabolites (M-III and M-IV) [90]. Similarly, luseogliflozin and tofogliflozin exhibited no relevant effect on pioglitazone pharmacokinetics, with most exposure ratios within the bioequivalence range of 0.80–1.25 [77,79].

Notably, one study reported an unexpected increase in pioglitazone exposure during initial co-administration with empagliflozin. However, this effect was not reproduced in a subsequent trial, suggesting methodological or interindividual variability rather than a true pharmacokinetic interaction [90].

Lobeglitazone has also been tested in combination with empagliflozin and dapagliflozin. These studies found no significant changes in the exposure levels of either drug, reinforcing the general safety of this combination [91,92]. In vitro findings suggest that lobeglitazone weakly interacts with CYP1A2, CYP2C9, CYP2C19, and MDR1, as well as OATP1B1 transporters [92]; however, these effects appear to be minimal in vivo.

In summary, based on current evidence, the combination of SGLT2 inhibitors and thiazolidinediones does not result in clinically meaningful pharmacokinetic interactions. The co-administration is well tolerated, with preserved drug exposure levels and metabolic profiles, supporting the continued use of these agents in combination regimens for T2DM, particularly in patients requiring multitargeted glycemic control strategies.

**Table 7:** Clinical drug interaction studies of SGLT2 inhibitors with thiazolidinedione.

Perpetrator		Victim		Subjects	GMR [C_max_, AUC (90% CI)] ^a^	Ref.
Drug	Dosing Regimen	Drug	Dosing Regimen			
SGLT2 inhibitors as perpetrators						
Dapagliflozin	50 mg	Pioglitazone	45 mg	HV	0.93 (0.75–1.15), 1.00 (0.90–1.13)	[75]
Dapagliflozin	10 mg/day, MD	Lobeglitazone	0.5 mg/day, MD	HV	0.97 (0.91–1.04), 0.97 (0.92–1.01)	[91]
Empagliflozin/metformin	25 mg/2000 mg/day, MD	Lobeglitazone	0.5 mg/day, MD	HV	1.08 (1.03–1.14), 0.98 (0.90–1.07)	[92]
Empagliflozin	25 mg/day, MD	Lobeglitazone	0.5 mg/day, MD	HV	0.93 (0.87–0.99), 0.93 (0.85–1.02)	[93]
Empagliflozin	10 mg	Pioglitazone	45 mg	HV	[M-III] ^c^ 0.97, 0.99 ^b^/ [M-IV] ^d^ 0.99, 0.99 ^b^/0.78, 0.84 ^b^	[90]
Empagliflozin	10 mg/day, MD	Pioglitazone	45 mg/day, MD	HV	[M-III] 0.82, 0.95 ^b^ [M-IV] 0.83, 0.94 ^b^/0.88, 0.88 ^b^	[90]
Empagliflozin	25 mg/day, MD	Pioglitazone	45 mg/day, MD	HV	[M-III] 0.96, 0.97 ^b^ [M-IV] 1.08, 1.02 ^b^/1.12, 1.03 ^b^	[90]
Empagliflozin	50 mg	Pioglitazone	45 mg	HV	[M-III] 1.02, 1.04 ^b^ [M-IV] 1.03, 1.03/0.84, 0.88 ^b^	[90]
Empagliflozin	50 mg/day, MD	Pioglitazone	45 mg/day, MD	HV	[M-III] 0.79, 0.93 ^b^ [M-IV] 0.80, 0.93 ^b^	[90]
Empagliflozin	50 mg/day, MD	Pioglitazone	45 mg/day, MD	HV	1.88 (1.66–2.12), 1.58 (1.48–1.69) [M-III] 1.22, 1.15 ^b^, [M-IV] 1.20, 1.15 ^b^	[90]
Luseogliflozin	5 mg	Pioglitazone	30 mg/day, MD	HV	0.88 (0.75, 1.05), 0.90 (0.77, 1.04) [M-III] 1.04 (0.97, 1.11), 1.01 (0.95, 1.07) [M-IV] 1.01 (0.95, 1.07), 1.03 (0.98, 1.09)	[77]
Ipragliflozin	150 mg, MD	Pioglitazone	30 mg	HV	0.99 (0.88–1.11), 1.02 (0.97–1.07)	[83]
Tofogliflozin	40 mg	Pioglitazone	45 mg	HV	1.14 (1.01–1.29), 1.08 (0.98–1.18) [M-III] 1.20 (1.07–1.35), 1.11 (1.02–1.21) [M-IV] 1.14 (1.03–1.27), 1.08 (0.99–1.18)	[79]
SGLT2 inhibitors as victims						
Lobeglitazone	0.5 mg/day, MD	Dapagliflozin	10 mg/day, MD	HV	0.92 (0.77–1.11), 0.99 (0.96–1.03)	[91]
Lobeglitazone	0.5 mg/day, MD	Empagliflozin/ metformin	25 mg/2000 mg/day, MD	HV	[Empagliflozin] 0.87 (0.78–0.97), 0.97 (0.93–1.00)	[92]
Lobeglitazone	0.5 mg/day, MD	Empagliflozin	25 mg/day, MD	HV	1.05 (0.96–1.15), 1.04 (0.95–1.11)	[93]
Pioglitazone	45 mg	Dapagliflozin	50 mg	HV	1.09 (1.00–1.18), 1.03 (0.98–1.08)	[75]
Pioglitazone	45 mg/day, MD	Empagliflozin	50 mg/day, MD	HV	0.93 (0.85–1.02), 1.00 (0.96–1.05)	[90]
Pioglitazone	30 mg/day, MD	Luseogliflozin	5 mg	HV	1.16 (1.04, 1.30), 0.94 (0.90, 0.98)	[77]
Pioglitazone	30 mg/day, MD	Ipragliflozin	150 mg	HV	0.94 (0.86–1.01), 1.00 (0.98–1.02)	[83]
Pioglitazone	45 mg	Tofogliflozin	40 mg	HV	1.04 (0.92–1.19), 1.01 (0.98–1.04)	[79]

HV, healthy volunteers; MD, multiple dosing; HV, healthy volunteers; IR, intermediate release; MD, multiple dosing. ^a^ Geometric mean ratio of C_max_ or AUC of the victim drug in the presence of the perpetrator to that in its absence. ^b^ Presented as arithmetic mean ratio. ^c^ Active metabolite of pioglitazone (keto pioglitazone). ^d^ Active metabolite of pioglitazone (hydroxy pioglitazone).

### 6.4. Interactions of SGLT2 Inhibitors with Other Class Drugs

Combination therapy of SGLT2 inhibitors with sulfonylureas (such as glimepiride or glyburide) has been examined in healthy volunteers, given the hypoglycemic risk of sulfonylureas (Table 8). These studies showed that SGLT2 inhibitors do not meaningfully affect sulfonylurea pharmacokinetics, nor do sulfonylureas affect SGLT2 inhibitors. Dapagliflozin (20–50 mg) co-administered with glimepiride (4 mg) resulted in virtually no change in dapagliflozin exposure and only a slight increase in glimepiride exposure [75]. In this study, glimepiride AUC during co-administration was approximately 11% higher than during glimepiride monotherapy and the 90% confidence interval upper limit was 1.29. This small AUC increase exceeded the typical cutoff of 1.25 but was not associated with any adverse effects or considered clinically significant. Ipragliflozin was also tested in combination with glimepiride and showed no significant interaction in either direction [83]. In a Japanese trial of tofogliflozin, co-administration with glimepiride did not affect tofogliflozin exposure and the C_max_ and AUC of glimepiride were virtually unchanged [79]. Similarly, henagliflozin did not significantly alter the pharmacokinetic profile of glimepiride, and a single dose of glimepiride had minimal effects on the levels of henagliflozin [94]. For glyburide, which is metabolized by CYP2C9 and CYP3A, no pharmacokinetic interaction with SGLT2 inhibitors has been observed either. A dedicated study of canagliflozin (200 mg) with low-dose glyburide (1.25 mg) found that co-administration did not affect the overall glyburide exposure or its active metabolite [74]. In particular, the C_max_ and AUC of glyburide in the presence of canagliflozin remained unchanged. The 4-trans-hydroxy and 3-cis-hydroxy glyburide metabolite levels were also unaffected. This confirms that mild inhibition of CYP2C9 by canagliflozin (IC_50_ = 80 μM) does not translate into a clinically important effect on glyburide metabolism, indicating that SGLT2 inhibitors can be safely added to sulfonylurea therapy without altering the pharmacokinetic or metabolic profile of sulfonylureas, although the additive pharmacodynamic effects on blood glucose and hypoglycemia risk still require monitoring.

Alpha-glucosidase inhibitors, such as miglitol and voglibose, act locally in the gut to delay carbohydrate absorption and are either minimally absorbed (voglibose) or absorbed and excreted unchanged (miglitol). Given their mechanism of action, significant metabolic drug interactions with SGLT2 inhibitors are not expected, as supported by clinical data. Trials in healthy volunteers and patients with T2DM found no meaningful pharmacokinetic interactions between SGLT2 inhibitors and alpha-glucosidase inhibitors. For example, a study in Japan reported that luseogliflozin (5 mg) combined with miglitol did not alter miglitol exposure, and the change in luseogliflozin C_max_ was minimal (a slight decrease that remained within bioequivalence limits) [77]. In this study, the geometric mean ratio (GMR) of luseogliflozin C_max_ with miglitol was modestly lower (GMR of C_max_: 0.85); however, this small change was not clinically meaningful and did not affect overall glucose-lowering efficacy. The slight reduction in the C_max_ of the SGLT2 inhibitor was plausibly due to delayed intestinal glucose absorption, which slowed the uptake rate of the drug; however, the total exposure (i.e., AUC) was essentially unaltered. Crucially, the changes remained within the 80–125% range, and no safety issues were noted. As voglibose has negligible systemic absorption, pharmacokinetic interaction studies have focused on whether ongoing voglibose therapy alters SGLT2 inhibitor absorption or disposition. In a study of Japanese patients with T2DM treated with stable voglibose (0.2 mg TID), a single dose of dapagliflozin (10 mg) was administered with and without voglibose to assess pharmacokinetic differences. The results showed that voglibose had no effect on the plasma profile of dapagliflozin; the AUC and C_max_ differed by <5% with and without voglibose [97]. No delay in dapagliflozin T_max_ or change in half-life was observed, indicating that slowing carbohydrate breakdown did not impede dapagliflozin absorption or elimination. Similarly, tofogliflozin showed no pharmacokinetic interaction with voglibose in healthy volunteers [79]. Overall, across SGLT2 inhibitors, co-administration with miglitol or voglibose showed no significant changes in the pharmacokinetic parameters of either agent. The absence of alpha-glucosidase inhibitor metabolism suggests that SGLT2 inhibitors have no enzymatic pathway to inhibit or induce, and neither drug is a known potent MDR1/OATP modulator that would affect the disposition of the other. At most, a minor interaction in absorption kinetics may occur (e.g., a slightly lower SGLT2 inhibitor C_max_ due to delayed glucose absorption in the gut); however, this interaction is not considered clinically significant.

### 6.5. Interactions of SGLT2 Inhibitors with Non-Antidiabetic Agents

Rifampin induces UGT enzymes [98], which are key metabolic pathways for several SGLT2 inhibitors, including canagliflozin, dapagliflozin, and ertugliflozin. In vitro studies consistently show that these agents rely predominantly on UGT-mediated glucuronidation for metabolic clearance. Consistent with these in vitro findings, clinical studies in healthy volunteers demonstrate that rifampin (600 mg daily for 5–7 days) decreases the AUC of canagliflozin by 51% [99] and of dapagliflozin by approximately 22% [100]. A similar decrease (39% decrease in AUC) was reported for ertugliflozin in combination with rifampin [101] (Table 9). Thus, when SGLT2 inhibitors are administered with rifampin or other strong UGT inducers, patients should be monitored for the loss of glycemic control. Interestingly, the co-administration of rifampin with empagliflozin resulted in an increase in systemic exposure. This observation is likely attributable to the minimal contribution of metabolism to empagliflozin clearance and the inhibitory effect of rifampin on hepatic uptake transporters, such as OATP1B1 and OATP1B3, which are involved in the hepatic disposition of empagliflozin [96].

The clinical relevance of UGT-mediated interactions is further supported by UGT inhibitors: probenecid increases canagliflozin exposure by 21% (AUC) [99], while mefenamic acid, a UGT1A9 inhibitor, increases the AUC of dapagliflozin by approximately 51% [100]. Although these increases remain below levels typically associated with toxicity or loss of therapeutic control [100], they underscore the importance of UGT pathways in the disposition of most SGLT2 inhibitors. Notably, because many SGLT2 inhibitors are metabolized through multiple UGT isoforms, the inhibition of a single enzyme does not usually result in excessive accumulation. This contrasts with agents such as remogliflozin, which is primarily cleared via CYP3A4 and shows a marked increase in exposure (up to 75%) when co-administered with ketoconazole, a strong CYP3A4 inhibitor [51], highlighting the necessity of understanding each agent’s dominant metabolic route when evaluating DDI risk.

In vitro studies show that SGLT2 inhibitors exhibit only weak inhibition of CYP3A4, with IC_50_ values far exceeding clinically relevant plasma concentrations (e.g., canagliflozin IC_50_ = 27 μM) and minimal interaction with hepatic uptake transporters such as OATP1B1. Consistent with these mechanistic findings, SGLT2 inhibitors demonstrate only minimal pharmacokinetic interactions with simvastatin in vivo. For instance, co-administration of canagliflozin (300 mg) with simvastatin (40 mg) resulted in small increases in simvastatin exposure (10% in C_max_ and 12% in AUC), and the simvastatin acid metabolite showed similarly modest changes (26% and 18% increases in C_max_ and AUC, respectively) [74]. Ertugliflozin likewise produced minor increases in simvastatin exposure (19% in C_max_ and 24% in AUC), which were not considered clinically meaningful [76]. These mild pharmacokinetic changes are consistent with the weak in vitro inhibition of CYP3A4 and OATP1B1 and do not result in meaningful pharmacodynamic effects. Reciprocally, simvastatin did not alter the pharmacokinetics of SGLT2 inhibitors. Overall, no dose adjustments are required when SGLT2 inhibitors are used in combination with simvastatin, reflecting the concordance between their in vitro interaction profiles and clinical outcomes.

Studies have indicated no significant interaction between SGLT2 inhibitors and warfarin. Dapagliflozin co-administered with warfarin did not affect the pharmacokinetics of either the S- or R-warfarin enantiomer (C_max_ and AUC changes were <10%) [102]. Importantly, the pharmacodynamic effect of warfarin [measured using the international normalized ratio (INR)] remained unchanged in the presence of dapagliflozin. Similarly, empagliflozin administered along with warfarin did not alter warfarin plasma levels or INR, confirming the lack of a clinically relevant interaction. Mechanistically, this was expected because SGLT2 inhibitors do not inhibit or induce CYP2C9, the primary enzyme involved in S-warfarin metabolism, at clinically relevant concentrations. Thus, SGLT2 inhibitors can be safely combined with warfarin; standard monitoring of INR is sufficient as per the usual warfarin management, and no interaction-related changes in the anticoagulant dose are required.

The concurrent use of SGLT2 inhibitors and antihypertensives did not show significant pharmacokinetic interactions. In healthy-volunteer studies, dapagliflozin (20 mg daily) had no effect on the plasma levels of valsartan (320 mg, an angiotensin receptor blocker), with C_max_ and AUC ratios of 1.06 versus valsartan alone [102]. Similarly, empagliflozin (25 mg daily) co-administered with ramipril [5 mg, an angiotensin-converting enzyme (ACE) inhibitor] did not significantly alter the pharmacokinetics of either drug, indicating no CYP-mediated interactions [103]. Similarly, empagliflozin exposure remained unchanged following verapamil (a calcium channel blocker and MDR1 inhibitor) co-administration, indicating that its clearance is not affected by CYP3A4/P-gp modulation [103]. However, additive blood pressure lowering was observed in patients receiving ramipril with empagliflozin, although this did not result in hypotension. Similarly, combined SGLT2 inhibitor and angiotensin II receptor blocker (ARB)/ACE inhibitor therapy is well tolerated, although clinicians should monitor for orthostatic hypotension or dizziness due to the combined antihypertensive effects. No special precautions or dose adjustments are necessary when initiating SGLT2 inhibitors in patients receiving ACE inhibitors, ARBs, or calcium channel blockers.

Co-administration of SGLT2 inhibitors with diuretics (thiazide or loop diuretics) has minimal impact on pharmacokinetics but can lead to additive diuretic effects. Canagliflozin and empagliflozin showed no significant pharmacokinetic changes when administered in combination with hydrochlorothiazide or loop diuretics. For instance, empagliflozin (25 mg) combined with hydrochlorothiazide (25 mg) or torasemide (5 mg) in patients with diabetes yielded plasma drug levels comparable to those observed with each drug alone (with combination-to-monotherapy AUC ratios of approximately 1.0) [104]. No alterations in plasma diuretic concentrations were observed, indicating the absence of CYP or transporter-based interactions. Pharmacodynamically, this combination may enhance natriuresis and reduces volume loss. In one study, dapagliflozin combined with bumetanide (1 mg) led to greater urinary sodium excretion than either agent alone, suggesting an additive diuretic effect [105]. Clinically, patients taking SGLT2 inhibitors and diuretics may experience a greater increase in urine output and a modest additional blood pressure reduction. Therefore, while no dosing changes are needed, monitoring volume status, blood pressure, and renal function is necessary, particularly at therapy initiation, to avoid excessive dehydration or hypotension.

**Table 9:** Clinical drug interaction study of SGLT2 inhibitors with non-antidiabetic drugs.

Perpetrator		Victim		Subject	GMR [C_max_, AUC (90% CI)] ^a^	Ref.
Drug	Dosing Regimen	Drug	Dosing Regimen			
SGLT2 inhibitors as perpetrators						
Canagliflozin	300 mg/day, MD	Tadalafil	20 mg	HV	1.10, 1.32 ^b^	[106]
Canagliflozin	300 mg/day, MD	Simvastatin	40 mg	HV	1.10 (0.91–1.31), 1.12 (0.94–1.33) [Simvastatin acid] 1.26 (1.10–1.45), 1.18 (1.03–1.35)	[74]
Canagliflozin	300 mg/day, MD	Hydrochlorothiazide	25 mg/day, MD	HV	0.94 (0.87–1.01), 0.99 (0.95–1.04)	[107]
Dapagliflozin	20 mg	Simvastatin	40 mg	HV	0.94 (0.82–1.07), 1.19 (1.01–1.40) [Simvastatin acid] 1.08 (0.93–1.25), 1.30 (1.15–1.47)	[102]
Dapagliflozin	20 mg	Valsartan	320 mg	HV	0.94 (0.76–1.16), 1.06 (0.87–1.30)	[102]
Dapagliflozin	20 mg on Day 1 and 10 mg on Day 2	Warfarin	25 mg	HV	[R-warfarin] 1.06 (1.00–1.15)/1.08 (1.03–1.12) [S-warfarin] 1.03 (0.99–1.12), 1.07 (1.01–1.14)	[102]
Dapagliflozin	20 mg on Day 1 and 10 mg on Day 2	Digoxin	0.25 mg	HV	0.99 (0.84–1.16), 1.00 (0.86–1.17)	[102]
Dapagliflozin	10 mg	Bumetanide	1 mg	HV	1.13 (0.98–1.31), 1.13 (0.99–1.30)	[105]
Ertugliflozin	15 mg	Simvastatin	40 mg	HV	1.19 (0.92–1.46), 1.24 (0.91–1.69) [Simvastatin acid] 1.16 (0.96–1.40), 1.31 (1.08–1.57)	[76]
Empagliflozin	25 mg/day, MD	Digoxin	0.5 mg	HV	1.14 (0.99–1.31), 1.06 (0.97–1.16)	[103]
Empagliflozin	25 mg/day, MD	Ramipril	2.5 mg/day, MD	HV	1.04 (0.90–1.20), 1.08 (1.01–1.16) [Ramiprilat] 0.98 (0.93–1.04), 0.99 (0.96–1.01)	[103]
Empagliflozin	25 mg/day, MD	Warfarin	25 mg	HV	[R-warfarin] 0.97 (0.91–1.05), 0.98 (0.95–1.02) [S-warfarin] 0.99 (0.92–1.06), 0.96 (0.93–0.98)	[108]
Empagliflozin	25 mg/day, MD	Hydrochlorothiazide	25 mg/day, MD	T2DM	1.02 (0.89–1.17), 0.96 (0.89–1.04)	[104]
Empagliflozin	25 mg/day, MD	Torasemide	5 mg/day, MD	T2DM	1.04 (0.94–1.16), 1.01 (0.99–1.04) [M1] 1.03 (0.94–1.12), 1.04 (1.00–1.09) [M3] 1.02 (0.98–1.07), 1.03 (0.96–1.11)	[104]
Empagliflozin	25 mg/day, MD	Ethinylestradiol/ levonorgestrel	30 μg/150 μg, MD	HV	[Ethinylestradiol] 0.99 (0.93–1.06), 1.03 (0.98–1.08) [Levonorgestrel] 1.06 (1.00–1.13), 1.02 (0.99–1.06)	[109]
Tofogliflozin	40 mg	Nateglinide	90 mg	HV	1.01 (0.84–1.22), 1.00 (0.961–1.05)	[79]
Enavogliflozin	2 mg/day, MD	Phentermine	37.5 mg/day, MD	HV	1.01, 0.94 ^b^	[81]
Henagliflozin	10 mg/day, MD	Warfarin	5 mg	HV	[R-warfarin] 1.15 (1.09–1.21), 1.21 (1.19–1.25) [S-warfarin] 1.14 (1.06–1.23), 1.21 (1.17–1.26)	[110]
Henagliflozin	10 mg/day, MD	Hydrochlorothiazide	25 mg/day MD	HV	1.24 (1.08, 1.43), 1.18 (1.15, 1.21)	[111]
Henagliflozin	10 mg/day, MD	Valsartan	160 mg	HV	0.83 (0.67, 1.02), 0.88 (0.76, 1.01)	[112]
SGLT2 inhibitors as victims						
Rifampin	600 mg/day, MD	Canagliflozin	300 mg	HV	0.72 (0.61–0.84), 0.49 (0.44–0.54) [M5] 1.61 (1.34–1.92), 1.04 (0.93–1.17) [M7] 1.31 (1.15–1.49), 0.68 (0.61–0.75)	[99]
Rifampin	600 mg/day, MD	Dapagliflozin	10 mg	HV	0.93 (0.78–1.11), 0.78 (0.73–0.83) [Glucuronide] 0.99, 0.86 ^b^	[100]
Rifampin	600 mg/day, MD	Ertugliflozin	15 mg	HV	0.85 (0.74–0.97), 0.61 (0.57–0.65)	[101]
Rifampin	600 mg	Empagliflozin	25 mg	HV	1.75 (1.60–1.91), 1.35 (1.29–1.41)	[96]
Probenecid	500 mg BID, MD	Canagliflozin	300 mg/day, MD	HV	1.13 (1.00, 1.28), 1.21 (1.16–1.25) [M5] 1.29 (1.16–1.44), 1.46 (1.35–1.59) [M7] 1.29 (1.20–1.37), 1.30 (1.26–1.34)	[99]
Cyclosporine	400 mg/day, MD	Canagliflozin	300 mg/day, MD	HV	1.01 (0.91–1.11), 1.23 (1.19–1.27)	[99]
Hydrochlorothiazide	25 mg/day, MD	Canagliflozin	300 mg/day, MD	HV	1.15 (1.06–1.25), 1.12 (1.08–1.17)	[107]
Simvastatin	40 mg	Dapagliflozin	20 mg	HV	0.98 (0.89–1.08), 0.98 (0.95–1.01)	[102]
Valsartan	320 mg	Dapagliflozin	20 mg	HV	0.88 (0.80–0.98), 1.02 (1.00–1.05)	[102]
Bumetanide	1 mg	Dapagliflozin	10 mg	HV	1.08 (0.95–1.22), 1.05 (0.99–1.11)	[105]
Sparsentan	800 mg/day, MD	Dapagliflozin	10 mg	HV	1.12, 1.07 ^b^ [Glucuronide] 0.90, 0.89 ^b^	[113]
Mefenamic acid	250 mg TID, MD	Dapagliflozin	10 mg	HV	1.13 (1.03–1.24)/1.51 (1.44–1.58) [Glucuronide] 0.56, 0.78 ^b^	[100]
Simvastatin	40 mg	Ertugliflozin	15 mg	HV	1.06, 1.03 ^b^	[76]
Verapamil	120 mg	Empagliflozin	25 mg	HV	0.92 (0.85–1.00), 1.03 (0.99–1.07)	[103]
Ramipril	2.5 mg/day, MD	Empagliflozin	25 mg/day, MD	HV	1.04 (0.98–1.12), 0.97 (0.93–1.00)	[103]
Warfarin	25 mg	Empagliflozin	25 mg	HV	1.01 (0.90–1.13), 1.01 (0.97–1.05)	[108]
Probenecid	500 mg BID, MD	Empagliflozin	25 mg/day, MD	HV	1.25 (1.13–1.38), 1.53 (1.46–1.60)	[96]
Hydrochlorothiazide	25 mg/day, MD	Empagliflozin	25 mg/day, MD	T2DM	1.03 (0.89–1.19), 1.0 (0.97–1.18)	[104]
Torasemide	5 mg/day, MD	Empagliflozin	25 mg/day, MD	T2DM	1.08 (0.98–1.18), 1.08 (1.00–1.16)	[104]
Nateglinide	90 mg	Tofogliflozin	40 mg	HV	0.96 (0.89–1.03), 1.08 (1.04–1.11)	[79]
Phentermine	37.5 mg/day, MD	Enavogliflozin	2 mg/day, MD	HV	0.98, 1 ^b^	[81]
Warfarin	5 mg	Henagliflozin	10 mg/day, MD	HV	1.02 (0.96–1.08), 1.02 (1.00–1.04)	[110]
Hydrochlorothiazide	25 mg/day, MD	Henagliflozin	10 mg/day, MD	HV	0.80 (0.72–0.91), 0.92 (0.85–1.00)	[111]
Valsartan	160 mg	Henagliflozin	10 mg/day, MD	HV	0.86 (0.76–0.98), 0.98 (0.95–1.01)	[112]
Ketoconazole	400 mg/day, MD	Remogliflozin etabonate	250 mg	HV	1.24 (0.92–1.68), 1.30 (1.04–1.62) [Remogliflozin] 1.32 (1.14–1.53), 1.75 (1.63–1.87)	[51]

BID, twice daily; HV, healthy volunteers; MD, multiple dosing; T2DM, type 2 diabetes mellitus; TID, three times daily. ^a^ Geometric mean ratio of C_max_ or AUC of the victim drug in the presence of the perpetrator to that in its absence. ^b^ Presented as arithmetic mean ratio.

The potential for DDIs between SGLT2 inhibitors and oral contraceptives has been of particular concern in premenopausal women with T2DM. To this end, empagliflozin has been studied for its potential interactions with combined oral contraceptives. In a phase I study involving healthy premenopausal women, the co-administration of empagliflozin (25 mg once daily) with a fixed-dose combination of ethinylestradiol and levonorgestrel showed no clinically relevant changes in the pharmacokinetics of either hormone. Both AUC and C_max_ values remained within the standard bioequivalence range of 0.8–1.25. These findings align with the mechanistic data indicating that empagliflozin does not inhibit or induce CYP3A4 or significantly affect drug transporters involved in hormone disposition [109].

Phosphodiesterase-5 inhibitors, such as tadalafil, are commonly co-administered with antihyperglycemic agents in patients with comorbid T2DM and erectile dysfunction. In a controlled clinical study involving healthy male volunteers, co-administration of canagliflozin (300 mg once daily for 5 days) with a single 20 mg dose of tadalafil resulted in only a slight increase in tadalafil exposure (both C_max_ and AUC increased by approximately 11%) and was not considered clinically significant [106]. The absence of additive hypotensive effects suggests that canagliflozin does not significantly inhibit or induce CYP3A4, the primary enzyme responsible for tadalafil metabolism. As both drug classes exert vasodilatory effects, monitoring blood pressure is prudent in practice; however, no contraindications exist, and no dosage adjustments are recommended during their concurrent use.

## 7. Challenges and Future Directions in Research for SGLT2 Inhibitor

In controlled studies, SGLT2 inhibitors have generally exhibited a low risk of clinically significant DDIs. However, several critical knowledge gaps exist in the literature (Table 10). Most interaction studies have been short-term trials conducted in healthy volunteers, leaving uncertainty about DDI risks during long-term, real-world use. A recent review highlighted the limited DDI data available for chronic use in heart failure patients [114]. In addition, data in special populations are lacking. For instance, while empagliflozin was recently approved for adolescent patients (≥10 years old), formal DDI studies in pediatric or older adult populations remain extremely limited. Similarly, more real-world evidence is needed to capture potential interactions under polypharmacy conditions typical of T2DM and cardiometabolic comorbidities. Case reports have already hinted at unanticipated issues; for example, SGLT2 inhibitors combined with statins may increase statin toxicity despite no obvious interactions in healthy volunteers [115,116]. Such discrepancies between controlled trials and clinical practice highlight the importance of post-marketing pharmacoepidemiologic studies to better understand the risk of DDIs.

Another emerging area of interest is the role of metabolites in the DDI profiles of SGLT2 inhibitors. These agents are often described as having no active metabolites. However, recent findings call for a more nuanced understanding. For example, sotagliflozin is extensively glucuronidated to its major metabolite M19 (sotagliflozin-3-O-glucuronide), which dominates plasma drug-related exposure. Although M19 shows minimal pharmacological activity at SGLT targets (>275-fold weaker than the parent drug), in vitro studies indicate that it can significantly influence pharmacokinetics by inducing or inhibiting CYP3A4, CYP2D6, and transporters such as OATP1B1/B3 and MRP2 [48]. This example illustrates how a nominally inactive metabolite could precipitate or contribute to DDIs. Similarly, the newly approved bexagliflozin is metabolized into several metabolites; one glucuronide (M5) accounts for approximately one-third of the parent AUC in humans [47]. Although all identified bexagliflozin metabolites possess less than 10% of the parent drug’s SGLT2-inhibiting potency, their potential to inhibit or induce metabolic enzymes or transporters has not been fully evaluated. Better characterization of such metabolites in terms of transporter inhibition or enzyme induction is required to complete the interaction profile of this drug class. Addressing this gap requires dedicated in vitro and in vivo studies focusing on metabolite-mediated modulation of pharmacokinetics.

In addition to pharmacokinetics, future DDI studies on SGLT2 inhibitors should include pharmacodynamic and system-level interactions. These drugs exert physiological effects such as osmotic diuresis, glycosuria, and shifts in ketone metabolism, which could synergize or conflict with co-medications. For instance, combining an SGLT2 inhibitor with insulin or sulfonylurea can enhance the risk of hypoglycemia, and adding an SGLT2 inhibitor to a loop or thiazide diuretic may exaggerate volume depletion and hypotensive effects. Indeed, the co-administration of empagliflozin with a diuretic has been shown to significantly increase urine output and the frequency of urination. Such interactions highlight the need for careful clinical monitoring, and further research should quantify these effects and establish appropriate management guidelines. Emerging concepts such as chronopharmacology also warrant investigation. For instance, it is unclear whether morning versus evening dosing alters interaction profiles or efficacy. Preliminary animal studies have suggested that the antihyperglycemic effect of dapagliflozin varies with the timing of administration [117]. However, translational data in humans are lacking. Additionally, the interplay between SGLT2 inhibitors and gut microbiota remains an open question. Although SGLT2 inhibitors do not exert primary pharmacologic effects in the gut, glucosuria-induced changes in glucose handling may modestly influence intestinal microbial composition [118]. Research is only beginning to explore whether these microbiota shifts could influence drug metabolism or nutrient–drug interactions. The investigation of gut microbiome-mediated DDIs could reveal subtle effects relevant to long-term SGLT2 inhibitor therapy.

Taken together, these considerations highlight the need for future research that systematically evaluates both parent compounds and their metabolites across key enzyme and transporter pathways. Particular attention is warranted for newer agents such as bexagliflozin, as well as for clinical scenarios involving polypharmacy, which are increasingly common in patients with diabetes and cardiometabolic comorbidities. Expanding the evidence base in clinically vulnerable populations—including individuals with hepatic or renal impairment, frail older adults, and pediatric patients—will also be important given their altered pharmacokinetics and heightened sensitivity to drug effects. Finally, strengthening the translational bridge between preclinical systems and clinical observations through humanized in vitro platforms, real-world pharmacoepidemiologic data, and iterative clinical validation will be essential for developing a more comprehensive and clinically actionable understanding of SGLT2 inhibitor interactions.

## 8. Conclusions

SGLT2 inhibitors provide substantial therapeutic benefits in T2DM and cardiometabolic diseases and generally exhibit favorable pharmacokinetic properties with a low risk of clinically meaningful DDIs. Their predominant glucuronidation pathways and minimal involvement of CYP enzymes contribute to their favorable interaction profile, including in polypharmacy settings. However, important knowledge gaps persist, particularly regarding metabolite-mediated interactions, long-term use in real-world populations, and data in vulnerable groups such as pediatric and older adult patients. As the therapeutic use of SGLT2 inhibitors continues to broaden, a more comprehensive understanding of their interaction potential will be essential to optimize safety and efficacy in real-world clinical practice.

## Figures and Tables

**Table 2:** In vitro pharmacokinetic characteristics of SGLT2 inhibitors and the metabolites.

Drug	BCS ^a^	Plasma Protein Binding	Metabolism			Transporter		Ref.
			Substrate	Inhibition (IC_50_)	Induction	Substrate	Inhibition (IC_50_)	
Canagliflozin	IV	98.2–99.0%	Extensive metabolism by UGT1A9/2B4 (M7/M5) Minor metabolism by CYP3A4/2D6 (M9)	CYP2B6/3A/2C8/2C9: 16/27/75/80 μM CYP1A2/2A6/2C19/2D6/2E1: >100 μM UGT1A1/1A6: 91/50 μM UGT1A9/2B7: >100 μM [M5, M7] CYP2B6/2C8: 55/64 μM, no inhibition of the other CYP isoforms (IC_50_ > 100 μM)	No induction of CYP3A4/2C9/2C19/1A2	Substrate of MDR1/BCRP/MRP2 Not substrate of OAT1/OAT3/OATP1B1/OATP1B3/OCT1/OCT2 [M5, M7] Not substrate of OAT1/OATP1B1/OCT1/OCT2	MDR1/MRP2/OATP1B1/OATP1B3/OCT1/OCT2: 19.3/21.5/24/32.6/5.2/44 μM No inhibition of BCRP at 10.5 μM No inhibition of OAT1/OAT3 [M5] No inhibition of OAT1/OATP1B1/OCT1/OCT2 at 100 μM [M7] Inhibition of OAT3 (54%) and OATP1B1 (65%) at 100 μM	[42,43]
Dapagliflozin	III	91%	Predominant metabolism by UGT1A9 (M15) Minor metabolism by CYP1A1/1A2/2A6/2C9/2D6/2E1/3A4	CYP1A2/2A6/2B6/2C8/2C9/2C19/2D6/3A4: >45 μM UGT1A1/1A9/1A10: 39–66 μM [M15] No inhibition of CYP 1A2/2C9/2C19/2D6/3A4	No induction of CYP 1A2/2B6/3A4 at 20 μM [M15] No induction of CYP 1A2/2B6/3A4 at 20 μM	Weak substrate of MDR1 [M15] Not substrate of OCT2/OAT1 [M15] Substrate of OAT3 (K_m_ 115 μM)	MDR1/OAT3: >57.6/33 μM No inhibition of OAT1/OCT2 [M15] MDR1/OAT3: >20.1/100 μM [M15] OAT1 inhibition (29%) at 100 μM [M15] No inhibition of OCT2	[44]
Empagliflozin	III	80.3–86.2%	Metabolism by UGT2B7/1A3/1A8/1A9 (less than 10% of total drug-related materials)	CYP1A2/2C9/2C19/3A4: >150 μM No TDI for CYP2C9/2D6/3A4 No inhibition of UGT1A1/1A3/1A8/1A9/2B7 [2-O, 3-O, and 6-O glucuronide] CYP1A2/2C9/2C19/2D6/3A4: >150 μM		Substrate of MDR1/BCRP/OAT3/OATP1B1/1B3 Not substrate of OAT1/OCT2	BCRP/MRP2/OAT1/OAT3/OCT2/OATP1B1/OATP1B3/OATP2B1: 114.1/1399/>1000/>295/>1000/71.8/58.6/45.2 μM	[45]
Ertugliflozin	I	93.6%	Predominant metabolism by UGT1A9/2B7 Minor metabolism by CYP2C8/3A	No inhibition of 1A2/2C9/2C19/2C8/2B6/2D6/3A4 No TDI of CYP3A4 UGT1A6/1A9/2B7: >39 μM No inhibition of UGT1A6/1A9/or 2B7 [3-O-ß, 2-O-ß glucuronide] No inhibition of UGT1A1/1A4/1A6/1A9/2B7	No induction of CYP 1A2/2B6/3A4 [3-O-ß, 2-O-ß glucuronide] No induction of CYP1A2/2B6/3A4	Substrate of MDR1/BCRP Not substrate of OAT1/OAT3/OCT1/OCT2/OATP1B1/OATP1B3	No inhibition of MDR1/OCT2/OAT1/OAT3/OATP1B1/OATP1B3 at clinically relevant concentrations [3-O-ß, 2-O-ß glucuronide] No inhibition of MDR1/OCT2/OAT1/OAT3/OATP1B1/OATP1B3 at clinically relevant concentrations	[46]
Bexagliflozin	III	90.9–93.0%	Predominant metabolism by UGT1A9 Minor metabolism by CYP3A	CYP2B6/2C8: >50 μM No inhibition of CYP1A2/2C9/2C19/2D6/3A4 at 50 μM	No induction CYP1A2/2B6/3A4 at 5 μM	Substrate of MDR1/BCRP Inconclusive for OATP1B1/1B3/OAT1/OAT3/OCT2/MATE1/MATE2-K [M5] Substrate of OATP1B1/OATP1B3/OAT3 [M5] Not substrate for BCRP/OAT1/OCT2/MATE1/MATE2-K	MDR1/BCRP/OATP1B1/OATP1B3/MATE1/MATE2-K: 3.7/79.2/34.8/57.5/45.5/>100 μM No inhibition of BSEP/OAT1/OAT3/OCT1/OCT2 [M5] MATE1/MATE2-K > 100 μM	[47]
Sotagliflozin	II	>91%	Predominant metabolism by UGT1A9 Minor metabolism by UGT1A1/2B7/CYP3A4	No inhibition of CYP1A2/2C9/2C19/2D6/3A4 [M19] Inhibition of CYP3A4/2D6	No induction of CYP1A2/2B6/3A4 [M19] Induction of CYP3A4	Substrate of OAT3/OATP1B1/OATP1B3 Not substrate of OAT1/OCT2 [M19] Substrate of BCRP/MRP2	Inhibition of MDR1/BCRP No inhibition of OCT1/OCT2/OAT1/OAT3/OATP1B1/OATP1B3 [M19] Inhibition of MRP2/OATP1B1/OATP1B3	[48]
Ipragliflozin	-	94.6–96.5%.	Predominant metabolism UGT2B7 Minor metabolism by UGT2B4/1A9	No or slight inhibition of CYP1A2/2A6/2B6/2C8/2C9/2C19/2D6/2E1/3A4/4A11 and UGT1A1/1A4/1A6/1A9/2B7	No induction of CYP1A2/3A4	Substrate of MDR1		[49]
Luseogliflozin	-	96.0–96.3%	Metabolism by CYP3A and UGT1A1/1A8/1A9	CYP2C19: 58.3 μM CYP1A2/2A6/2B6/28/2C9/2C19/2D6/2E1/3A4: >100 μM	No induction of CYP1A2/2B6 CYP3A4 3.5-fold increase in CYP3A4 activity at 10 μM	Substrate of MDR1 Not substrate of BCRP/OATP1B1/OATP1B3/OAT1/OAT3/OCT2	OATP1B3: 93.1 μM MDR1/BCRP/OATP1B1/OCT2/OAT1/OAT3: >100 μM	[50]
Remogliflozin etabonate	-	Remgliflozin: 65%	Metabolism to remogliflozin by esterase Remogliflozin: CYP3A4 (<50%) and other enzyme pathways (CYP450s, UGTs/glucosidases)					[51]
Tofogliflozin	-	82.3–82.6%	CYP2C8/3A4/3A5	CYP1A2/2B6/2C8/2C9/2C19/2D6/3A: >50 μM No TDI for CYP1A2/2C9/3A [M1] CYP2C19: 27.1 μM [M1] CYP1A2/2B6/2C8/2C9/2D6/3A: >50 μM [M1] No TDI for CYP1A2/2C9/3A	No induction of CYP1A2/3A4			[52]
Enavogliflozin	-	98.50%	Metabolism by CYP3A4 Minor metabolism by UGT1A4/1A9/2B7	No inhibition of CYP1A2/2A6/2B6/2C8/2C9/2C19/2D6/3A4 and UGT1A1/1A3/1A4/1A6/1A9/2B7 [M1] No inhibition of CYP1A2/2A6/2B6/2C8/2C9/2C19/2D6/3A4 and UGT1A1/1A3/1A4/1A6/1A9/2B7	No induction of CYP/1A2/2B6/3A4 [M1] No induction of CYP/1A2/2B6/3A4	Substrate of MDR1/OAT1/OAT3/OCT1/OCT2/OATP1B1/OATP1B3	No inhibition of OCT1/OCT2/OAT1/BCRP [M1] No inhibition of MDR1/BCRP/OAT1/OAT3/OATP1B1/NTCP	[53,54]

^a^ Biopharmaceutics Classification System (BCS) categorizes drugs into four classes based on their solubility and permeability: Class I (high solubility, high permeability), Class II (low solubility, high permeability), Class III (high solubility, low permeability), and Class IV (low solubility, low permeability); NA, not available; TDI, time-dependent inhibition.

**Table 3:** Clinical pharmacokinetic characteristics of SGLT2 inhibitors.

Drug	Oral F	T_max_	V_d_	Elimination	Dose-Proportionality	Effect on Pharmacokinetics				Ref.
						Food	Renal Impairment	Hepatic Impairment	Other Factors	
Canagliflozin	65%	1–2 h	119 L	CL 192 mL/min 41.5, 7.0, 3.2% into feces (canagliflozin, a hydroxylated metabolite, and an O-glucuronide metabolite 33% into urine (30.5% as O-glucuronide metabolite, <1% as canagliflozin)	Dose proportional increase within 50–300 mg	No effect	Mild, moderate, and severe: ↑ 15%, ↑ 29%, and ↑ 53% in AUC	Mild: ↑ 7% in C_max_ ↑ 11% in AUC Moderate: ↑ 10% in AUC	No effect of age, body weight, gender, and race	[43]
Dapagliflozin	78%	1 h	118 L	CL 207 mL/min 75% into urine (<2% as dapagliflozin) 21% into feces (~15% as dapagliflozin)	Dose proportional increase in the therapeutic dose range	↓ 50% in C_max_ No change in AUC Delayed T_max_ by 1 h	Mild, moderate, and severe: ↑ 45%, ↑ 100%, and ↑ 200% in systemic exposure	Mild and moderate: ↑ 12% in C_max_ and ↑ 36% in AUC Severe: ↑ 40% and ↑ 67% in C_max_ and AUC	No effect of age, body weight, gender, and race	[44]
Empagliflozin	78%	1.5 h	73.8 L (V_d_/F)	CL/F 177 mL/min Metabolism: <10% 41.2% into feces (majority is empagliflozin) 54.4% into urine (~50% as empagliflozin)	Dose proportional increase within 50–300 mg	↓ 16% in C_max_ and ↓ 37% in AUC	Mild, moderate, and severe: ↑ 20% in C_max_ and ↑ 18%, ↑ 20%, 66% in AUC	Mild, moderate, and severe: ↑ 4%, ↑ 23%, and ↑ 48% in C_max_ and ↑ 23%, ↑ 47%, and ↑ 75% in AUC	No effect of age, body weight, gender, and race	[45,55]
Ertugliflozin	100%	1 h	85.5 L	CL 187 mL/min 40.9% into feces (33.8% as ertugliflozin) 50.2% into urine (1.5% as ertugliflozin)	Dose proportional increase within 0.5–300 mg (single dose) and 1–100 mg (multiple dose)	↓ 29% in C_max_ No change in AUC Delayed T_max_ by 1 h	Mild, moderate, and severe: ↑ 60%, ↑ 70%, ↑ 60% in AUC	Moderate: ↑ 21% and ↑ 13% in C_max_ and AUC	No effect of age, body weight, gender, and race	[46]
Bexagliflozin	-	2–4h	262 L (V_d_/F)	CL/F 318 mL/min 51.1% into feces (28.7% as bexagliflozin) 40.5% into urine (1.5% as bexagliflozin)	Dose proportional increase within 3–90 mg (single dose)	↑ 31% in C_max_ and ↑ 10% in AUC Delayed T_max_ to 5 h	Mild, moderate, and severe: ↑ 7%, ↑ 34%, and ↑ 54% in AUC	Moderate: ↑ 6.3% and ↑ 28% in C_max_ and AUC	No effect of age, body weight, gender, and race	[47,56]
Sotagliflozin	25%	1.25–4h	9392 L (V_d_/F)	CL/F 4300–6166 mL/min 57% into urine (33% as 3-O-glucuronide) 37% into feces (23% as sotagliflozin)	Dose proportional increase in the therapeutic dose range of 200 mg to 400 mg	↑ 149% in C_max_ and ↑ 50% in AUC	Mild and moderate: ↑ 70% and ↑ 170% in AUC	Mild: No increase in AUC Moderate and severe: ↑ 3–6-fold in AUC	No effect of age, body weight, gender, and race	[48]

AUC, area under the curve; CL, clearance; C_max_, maximum concentration; F, bioavailability; T2DM, type 2 diabetic mellitus; T_max_, time to reach maximum concentration; V_d_, volume of distribution.; ↓, decrease; ↑, increase.

**Table 8:** Clinical drug interaction studies of SGLT2 inhibitors with other antidiabetic drugs.

Perpetrator		Victim		Subject	GMR [C_max_, AUC (90% CI)] ^a^	Ref.
Drug	Dosing Regimen	Drug	Dosing Regimen			
SGLT2 inhibitors as perpetrators						
Canagliflozin	200 mg/day, MD	Glyburide	1.25 mg	HV	0.93 (0.8–1.01), 1.02 (0.98–1.07)	[74]
Dapagliflozin	20 mg	Glimepiride	4 mg	HV	1.04 (0.91–1.20), 1.13 (1.00–1.29)	[75]
Ertugliflozin	15 mg	Glimepiride	1 mg	HV	0.97, 1.19 ^b^	[76]
Luseogliflozin	5 mg	Glimepiride	1 mg	HV	1.03 (0.95–1.12), 1.07 (1.04–1.10)	[77]
Ipragliflozin	150 mg, MD	Glimepiride	1 mg	HV	1.10 (1.02–1.19), 1.05 (1.01–1.09)	[83]
Tofogliflozin	40 mg	Glimepiride	1 mg	HV	0.99 (0.91–1.08), 1.09 (1.06–1.13)	[79]
Henagliflozin	10 mg/day, MD	Glimepiride	2 mg	HV	1.00 (0.88–1.13), 0.91 (0.84–0.99)	[94]
Luseogliflozin	5 mg	Miglitol	50 mg	HV	1.02 (0.92–1.14), 1.04 (0.94–1.16)	[77]
Ipragliflozin	100 mg	Miglitol	75 mg	HV	0.76 (0.67, 0.86), 0.80 (0.72, 0.88)	[95]
Tofogliflozin	40 mg	Miglitol	75 m	HV	1.04 (0.91–1.19), 1.06 (0.91–1.24)	[79]
SGLT2 inhibitors as victims						
Gemfibrozil	600 mg BID, MD	Empagliflozin	25 mg/day, MD	HV	1.15 (1.06–1.25), 1.58 (1.51–1.65)	[96]
Glimepiride	4 mg	Dapagliflozin	20 mg	HV	0.93 (0.85–1.02), 1.00 (0.94–1.05)	[75]
Glimepiride	1 mg	Ertugliflozin	15 mg	HV	1.01, 1.04 ^b^	[76]
Glimepiride	1 mg	Luseogliflozin	5 mg	HV	1.00 (0.90–1.12), 1.00 (0.98–1.03)	[77]
Glimepiride	1 mg/day, MD	Ipragliflozin	150 mg	HV	0.97 (0.89–1.06), 0.99 (0.97–1.02)	[83]
Glimepiride	1 mg	Tofogliflozin	40 m	HV	1.09 (0.96–1.22), 1.01 (0.97–1.06)	[79]
Glimepiride	2 mg	Henagliflozin	10 mg/day, MD	HV	1.00 (0.93–1.08), 1.00 (0.98–1.02)	[94]
Voglibose	0.2 mg TID, MD	Dapagliflozin	10 mg	T2DM	1.04 (0.90–1.20), 1.01 (0.95–1.07)	[97]
Voglibose	0.6 mg/day, MD	Luseogliflozin	5 mg	HV	1.09 (0.98–1.21), 1.02 (0.99–1.06)	[77]
Voglibose	0.3 mg	Tofogliflozin	40 mg	HV	1.03 (0.93–1.13), 1.00 (0.96–1.04)	[79]
Miglitol	50 mg	Luseogliflozin	5 mg	HV	0.85 (0.76–0.95), 0.95 (0.93–0.98)	[77]
Miglitol	75 mg	Ipragliflozin	100 mg	HV	1.03 (0.94, 1.13), 1.02 (0.99, 1.04)	[95]
Miglitol	75 mg	Tofogliflozin	40 mg	HV	0.93 (0.89–0.98), 0.97 (0.95–1.00)	[79]

BID, twice daily; HV, healthy volunteers; MD, multiple dosing; T2DM, type 2 diabetes mellitus; TID, three times daily. ^a^ Geometric mean ratio of C_max_ or AUC of the victim drug in the presence of the perpetrator to that in its absence. ^b^ Presented as arithmetic mean ratio.

**Table 10:** Current limitations in pharmacokinetic information for SGLT2 inhibitors and suggestions for future research.

Current Limitations	Future Research
Evidence mainly from short-term healthy volunteer studies	Long-term real-world PK/PD studies in T2DM, heart failure, CKD Pharmacoepidemiologic DDI surveillance
Lack of data in special populations	Dedicated PK/DDI studies in population subgroups PopPK/PBPK modeling to predict DDIs when trials are not feasible
Underexplored metabolite-mediated interactions	Systematic metabolite screening for CYP/UGT/transporter effects Integration of metabolite PK into PBPK models
Incomplete transporter characterization	Expanded transporter liability profiling Mechanistic IVIVE for transporter-based DDIs
Pharmacodynamic/system-level interactions not adequately quantified	Controlled studies on diuretic synergy, hypoglycemia risk, ketone shifts Development of clinical monitoring and dose-adjustment strategies
Chronopharmacology not incorporated	Human time-of-day PK/PD studies Chronopharmacology modeling to assess DDI sensitivity
Uncertain role of gut microbiome in DDI modulation	Metagenomics-integrated PK studies Investigation of microbiome–drug–DDI pathways
Limited translational models bridging in vitro to clinical outcomes	Humanized organoid and microphysiological platforms Combined IVIVE + real-world validation frameworks

CKD, chronic kidney disease; CYP, cytochrome P450; DDI, drug interactions; IVIVE, in vitro-in vivo extrapolation; PD, pharmacodynamics; PK, pharmacokinetics; PopPK, population pharmacokinetics; T2DM, type 2 diabetes mellitus; UGT, uridine diphosphate glucuronosyltransferases.

## Data Availability

No new data were created or analyzed in this study. Data sharing is not applicable to this article.

## References

[1] Sun H., Saeedi P., Karuranga S., Pinkepank M., Ogurtsova K., Duncan B.B., Stein C., Basit A., Chan J.C., Mbanya J.C. (2022). IDF Diabetes Atlas: Global, regional and country-level diabetes prevalence estimates for 2021 and projections for 2045. Diabetes Res. Clin. Pract..

[2] Nichols G.A., Amitay E.L., Chatterjee S., Steubl D. (2023). The Bidirectional Association of Chronic Kidney Disease, Type 2 Diabetes, Atherosclerotic Cardiovascular Disease, and Heart Failure: The Cardio–Renal–Metabolic Syndrome. Metab. Syndr. Relat. Disord..

[3] Hunter R.W., Hughey C.C., Lantier L., Sundelin E.I., Peggie M., Zeqiraj E., Sicheri F., Jessen N., Wasserman D.H., Sakamoto K. (2018). Metformin reduces liver glucose production by inhibition of fructose-1-6-bisphosphatase. Nat. Med..

[4] Foretz M., Guigas B., Viollet B. (2023). Metformin: Update on mechanisms of action and repurposing potential. Nat. Rev. Endocrinol..

[5] Bailey C.J. (2024). Metformin: Therapeutic profile in the treatment of type 2 diabetes. Diabetes Obes. Metab..

[6] Guardado-Mendoza R., Prioletta A., Jiménez-Ceja L.M., Sosale A., Folli F. (2013). The role of nateglinide and repaglinide, derivatives of meglitinide, in the treatment of type 2 diabetes mellitus. Arch. Med. Sci..

[7] DiNicolantonio J.J., Bhutani J., O’Keefe J.H. (2015). Acarbose: Safe and effective for lowering postprandial hyperglycaemia and improving cardiovascular outcomes. Open Heart.

[8] Ko K.D., Kim K.K., Lee K.R. (2017). Does Weight Gain Associated with Thiazolidinedione Use Negatively Affect Cardiometabolic Health?. J. Obes. Metab. Syndr..

[9] Ahrén B. (2019). DPP-4 inhibition and the path to clinical proof. Front. Endocrinol..

[10] Mullur N., Morissette A., Morrow N.M., Mulvihill E.E. (2024). GLP-1 receptor agonist-based therapies and cardiovascular risk: A review of mechanisms. J. Endocrinol..

[11] Chao E.C., Henry R.R. (2010). SGLT2 inhibition-a novel strategy for diabetes treatment. Nat. Rev. Drug Discov..

[12] Pinto L.C., Rados D.V., Remonti L.R., Viana M.V., Leitão C.B., Gross J.L. (2022). Dose-ranging effects of SGLT2 inhibitors in patients with type 2 diabetes: A systematic review and meta-analysis. Arch. Endocrinol. Metab..

[13] Pinto L.C., Rados D.V., Remonti L.R., Kramer C.K., Leitao C.B., Gross J.L. (2015). Efficacy of SGLT2 inhibitors in glycemic control, weight loss and blood pressure reduction: A systematic review and meta-analysis. Diabetol. Metab. Syndr..

[14] Zinman B., Wanner C., Lachin J.M., Fitchett D., Bluhmki E., Hantel S., Mattheus M., Devins T., Johansen O.E., Woerle H.J. (2025). Empagliflozin, Cardiovascular Outcomes, and Mortality in Type 2 Diabetes. N. Engl. J. Med..

[15] Guthrie R. (2018). Canagliflozin and cardiovascular and renal events in type 2 diabetes. Postgrad. Med..

[16] Wiviott S.D., Raz I., Bonaca M.P., Mosenzon O., Kato E.T., Cahn A., Silverman M.G., Zelniker T.A., Kuder J.F., Murphy S.A. (2019). Dapagliflozin and Cardiovascular Outcomes in Type 2 Diabetes. N. Engl. J. Med..

[17] Heerspink H.J.L., Stefánsson B.V., Correa-Rotter R., Chertow G.M., Greene T., Hou F.-F., Mann J.F.E., McMurray J.J.V., Lindberg M., Rossing P. (2020). Dapagliflozin in Patients with Chronic Kidney Disease. N. Engl. J. Med..

[18] Tiwari D., Loh W.J., Aw T.C. (2025). Updates from the 2025 American Diabetes Association guidelines on standards of medical care in diabetes. Explor. Endocr. Metab. Dis..

[19] Marx N., Schütt K., Müller-Wieland D., Di Angelantonio E., Herrington W.G., Ajjan R.A., Kautzky-Willer A., Rocca B., Sattar N., Fauchier L. (2025). Key priorities for the implementation of the 2023 ESC Guidelines for the management of cardiovascular disease in patients with diabetes in low-resource settings. Eur. Heart J. Qual. Care Clin. Outcomes.

[20] McDonagh T.A., Metra M., Adamo M., Gardner R.S., Baumbach A., Böhm M., Burri H., Butler J., Čelutkienė J., Chioncel O. (2021). 2021 ESC Guidelines for the diagnosis and treatment of acute and chronic heart failure. Eur. Heart J..

[21] Rossing P., Caramori M.L., Chan J.C.N., Heerspink H.J.L., Hurst C., Khunti K., Liew A., Michos E.D., Navaneethan S.D., Olowu W.A. (2022). KDIGO 2022 Clinical Practice Guideline for Diabetes Management in Chronic Kidney Disease. Kidney Int..

[22] Katsiki N., Ferrannini E., Mantzoros C. (2020). New American Diabetes Association (ADA)/European Association for the Study of Diabetes (EASD) guidelines for the pharmacotherapy of type 2 diabetes: Placing them into a practicing physician’s perspective. Metab. Clin. Exp..

[23] Gallwitz B. (2019). Clinical Use of DPP-4 Inhibitors. Front. Endocrinol..

[24] United States Food and Drug Administration (2024). Prescribing Information of Invokana (Canagliflozin). https://www.accessdata.fda.gov/drugsatfda_docs/label/2024/204042s043lbl.pdf.

[25] (2024). Prescribing Information of Farxiga (Dapagliflozin). https://www.accessdata.fda.gov/drugsatfda_docs/label/2024/202293s031lbl.pdf.

[26] (2023). Prescribing Information of Jardiance (Empagliflozin). https://www.accessdata.fda.gov/drugsatfda_docs/label/2023/204629s040lbl.pdf.

[27] (2024). Prescribing Information of Steglatro (Ertugliflozin). https://www.accessdata.fda.gov/drugsatfda_docs/label/2024/209803s008lbledt.pdf.

[28] (2023). Prescribing Information of Brenzavvy (Bexagliflozin). https://www.accessdata.fda.gov/drugsatfda_docs/label/2023/214373s001lbl.pdf.

[29] (2023). Prescribing Information of Inpefa (Sotagliflozin). https://www.accessdata.fda.gov/drugsatfda_docs/label/2023/216203s000lbl.pdf.

[30] Poole R.M., Dungo R.T. (2014). Ipragliflozin: First global approval. Drugs.

[31] Markham A., Elkinson S. (2014). Luseogliflozin: First global approval. Drugs.

[32] Poole R.M., Prossler J.E. (2014). Tofogliflozin: First global approval. Drugs.

[33] Markham A. (2019). Remogliflozin etabonate: First global approval. Drugs.

[34] Gao L., Cheng Z., Su B., Su X., Song W., Guo Y., Liao L., Chen X., Li J., Tan X. (2023). Efficacy and safety of janagliflozin as add-on therapy to metformin in Chinese patients with type 2 diabetes inadequately controlled with metformin alone: A multicentre, randomized, double-blind, placebo-controlled, phase 3 trial. Diabetes Obes. Metab..

[35] Zhang Y., Liu Y., Yu C., Wang Y., Zhan Y., Liu H., Zou J.J., Jia J.Y., Chen Q., Zhong D.F. (2021). Tolerability, pharmacokinetic, and pharmacodynamic profiles of henagliflozin, a novel selective inhibitor of sodium-glucose cotransporter 2, in healthy subjects following single-and multiple-dose administration. Clin. Ther..

[36] Filippas-Ntekouan S., Filippatos T.D., Elisaf M.S. (2018). SGLT2 inhibitors: Are they safe?. Postgrad. Med..

[37] Unnikrishnan A.G., Kalra S., Purandare V., Vasnawala H. (2018). Genital Infections with Sodium Glucose Cotransporter-2 Inhibitors: Occurrence and Management in Patients with Type 2 Diabetes Mellitus. Indian. J. Endocrinol. Metab..

[38] Bazoukis G., Papadatos S.S., Thomopoulos C., Tse G., Cheilidis S., Tsioufis K., Farmakis D. (2021). Impact of SGLT2 inhibitors on major clinical events and safety outcomes in heart failure patients: A meta-analysis of randomized clinical trials. J. Geriatr. Cardiol..

[39] Wilding J. (2019). SGLT2 inhibitors and urinary tract infections. Nat. Rev. Endocrinol..

[40] (2022). FDA Drug Safety Communication:FDA Revises Labels of SGLT2 Inhibitors for Diabetes to Include Warnings About Too Much Acid in the Blood and Serious Urinary Tract Infections. https://www.fda.gov/drugs/drug-safety-and-availability/fda-revises-labels-sglt2-inhibitors-diabetes-include-warnings-about-too-much-acid-blood-and-serious.

[41] SGLT2 Inhibitors—Referral 2016. https://www.ema.europa.eu/en/medicines/human/referrals/sglt2-inhibitors.

[42] Mamidi R.N.V.S., Dallas S., Sensenhauser C., Lim H.K., Scheers E., Verboven P., Cuyckens F., Leclercq L., Evans D.C., Kelley M.F. (2017). In vitro and physiologically-based pharmacokinetic based assessment of drug–drug interaction potential of canagliflozin. Br. J. Clin. Pharmacol..

[43] FDA Center for Drug Evaluation and Research (2013). Clinical Pharmacology and Biopharmaceutics Review (Canagliflozin). https://www.accessdata.fda.gov/drugsatfda_docs/nda/2013/204042Orig1s000ClinPharmR.pdf.

[44] FDA Center for Drug Evaluation and Research (2014). Clinical Pharmacology and Biopharmaceutics Review (Dapagliflozin). https://www.accessdata.fda.gov/drugsatfda_docs/nda/2014/202293Orig1s000ClinPharmR.pdf.

[45] FDA Center for Drug Evaluation and Research (2014). Clinical Pharmacology and Biopharmaceutics Review (Empagliflozin). https://www.accessdata.fda.gov/drugsatfda_docs/nda/2014/204629Orig1s000ClinPharmR.pdf.

[46] FDA Center for Drug Evaluation and Research (2017). Clinical Pharmacology and Biopharmaceutics Review (Ertugliflozin). https://www.accessdata.fda.gov/drugsatfda_docs/nda/2017/209803,209805,209806Orig1s000ClinPharmR.pdf.

[47] FDA Center for Drug Evaluation and Research (2023). Integrated Review (Bexagliflozin). https://www.accessdata.fda.gov/drugsatfda_docs/nda/2023/214373Orig1s000IntegratedR.pdf.

[48] FDA Center for Drug Evaluation and Research (2023). Integrated Review (Sotagliflozin). https://www.accessdata.fda.gov/drugsatfda_docs/nda/2023/216203Orig1s000IntegratedR.pdf.

[49] Ministry of Food and Drug Safety, Astellas Pharma Korea (2020). Ipragliflozin [Package Insert]. https://nedrug.mfds.go.kr/pbp/CCBBB01/getItemDetail?itemSeq=201404160.

[50] Chino Y., Hasegawa M., Fukasawa Y., Mano Y., Bando K., Miyata A., Nakai Y., Endo H., Yamaguchi J.I. (2017). In vitro evaluation of potential drug interactions mediated by cytochrome P450 and transporters for luseogliflozin, an SGLT2 inhibitor. Xenobiotica.

[51] Sigafoos J.F., Bowers G.D., Castellino S., Culp A.G., Wagner D.S., Reese M.J., Humphreys J.E., Hussey E.K., Semmes R.L.O., Kapur A. (2012). Assessment of the drug interaction risk for remogliflozin etabonate, a sodium-dependent glucose cotransporter-2 inhibitor: Evidence from in vitro, human mass balance, and ketoconazole interaction studies. Drug Metab. Dispos..

[52] Kobayashi K., Toyoda M., Hatori N. (2019). Clinical comparison of tofogliflozin and empagliflozin based on an analysis of 24-h accumulated urine in Japanese patients with type 2 diabetes mellitus. Obes. Med..

[53] Kim M.-S., Song Y.-K., Choi J.-S., Ji H.Y., Yang E., Park J.S., Kim H.S., Kim M.-J., Cho I.-K., Chung S.-J. (2023). Physiologically Based Pharmacokinetic Modelling to Predict Pharmacokinetics of Enavogliflozin, a Sodium-Dependent Glucose Transporter 2 Inhibitor, in Humans. Pharmaceutics.

[54] Choi M.-K., Nam S.J., Ji H.-Y., Park M.J., Choi J.-S., Song I.-S. (2020). Comparative pharmacokinetics and pharmacodynamics of a novel sodium-glucose cotransporter 2 inhibitor, DWP16001, with dapagliflozin and ipragliflozin. Pharmaceutics.

[55] Ndefo U.A., Anidiobi N.O., Basheer E., Eaton A.T. (2015). Empagliflozin (Jardiance): A Novel SGLT2 Inhibitor for the Treatment of Type-2 Diabetes. Pharm. Ther..

[56] Bassett R.L., Gallo G., Le K.-P.N., Volino L.R. (2024). Bexagliflozin: A comprehensive review of a recently approved SGLT2 inhibitor for the treatment of type 2 diabetes mellitus. Med. Chem. Res..

[57] Rodighiero V. (1989). Effects of cardiovascular disease on pharmacokinetics. Cardiovasc. Drugs Ther..

[58] Sundaram V., Fang J.C. (2016). Gastrointestinal and Liver Issues in Heart Failure. Circulation.

[59] Mangoni A.A., Jarmuzewska E.A. (2019). The influence of heart failure on the pharmacokinetics of cardiovascular and non-cardiovascular drugs: A critical appraisal of the evidence. Br. J. Clin. Pharmacol..

[60] Melin J., Parkinson J., Hamrén B., Penland R.C., Boulton D.W., Tang W. (2024). Dapagliflozin pharmacokinetics is similar between patients with heart failure with reduced ejection fraction and patients with type 2 diabetes mellitus. Br. J. Clin. Pharmacol..

[61] Rascher J., Cotton D., Haertter S., Brueckmann M. (2024). Clinical pharmacokinetics and pharmacodynamics of empagliflozin in patients with heart failure. Br. J. Clin. Pharmacol..

[62] He X., Li Y., Li Y., Guo C., Fu Y., Xun X., Wang Z., Dong Z. (2023). In vivo assessment of the pharmacokinetic interactions between donafenib and dapagliflozin, donafenib and canagliflozin in rats. Biomed. Pharmacother..

[63] Han D.-G., Yun H., Yoon I.-S. (2019). A novel high-performance liquid chromatographic method combined with fluorescence detection for determination of ertugliflozin in rat plasma: Assessment of pharmacokinetic drug interaction potential of ertugliflozin with mefenamic acid and ketoconazole. J. Chromatogr. B.

[64] Cui Y., Li Y., Guo C., Li Y., Ma Y., Dong Z. (2022). Pharmacokinetic interactions between canagliflozin and sorafenib or lenvatinib in rats. Molecules.

[65] Kandukoori N.R., Mandava K. (2023). Study on consequences for interaction of myricetin with canagliflozin: A special attention to pharmacokinetics and pharmacodynamics of drug. Pharm. Sci. Asia.

[66] He X., Li Y., Ma Y., Fu Y., Xun X., Cui Y., Dong Z. (2022). Development of UPLC-MS/MS method to study the pharmacokinetic interaction between sorafenib and dapagliflozin in rats. Molecules.

[67] He X., Li Y., Ma Y., Fu Y., Xun X., Dong Z. (2023). Pharmacokinetic interaction study between sorafenib and dapagliflozin in rats. Chinese J. Clin. Pharmacol. Ther..

[68] Wang L., Liang B., Teng Y., Zhang C., Zhang Y., Zhang Z., Zhang A., Dong S., Fan H. (2024). Assessment of drug–drug interaction of dapagliflozin with LCZ696 based on an LC–MS/MS method. Biomed. Chromatogr..

[69] Alhazzani K., Alanazi A.Z., Mostafa A.M., Barker J., El-Wekil M.M., Ali A.-M.B.H. (2023). A novel microextraction technique aided by air agitation using a natural hydrophobic deep eutectic solvent for the extraction of fluvastatin and empagliflozin from plasma samples: Application to pharmacokinetic and drug–drug interaction study. RSC Adv..

[70] Nanjappan S.K., Somabattini R.A., Ravichandiran V. (2022). Investigation of the effect of Acai berry on the pharmacokinetics of Atorvastatin, Alogliptin and Empagliflozin: A herb–drug interaction study. J. Pharm. Pharmacol..

[71] Abu Dayyih W., Zakaraya Z., Hailat M., Al-Tawarah N.M., Alkharabsheh S., Nadher H.K., Hailat Z., Alarman S.M., Khaleel A., Awad R. (2024). The Validation and Determination of Empagliflozin Concentration in the Presence of Grapefruit Juice Using HPLC for Pharmacokinetic Applications. Molecules.

[72] Meesa M., Yellu N.R. (2023). Impact of Sinapic acid on Ertugliflozin Pharmacokinetics and Pharmacodynamics in Type-2 Diabetic Rats. J. Young Pharm..

[73] Mizuno-Yasuhira A., Nakai Y., Gunji E., Uchida S., Takahashi T., Kinoshita K., Jingu S., Sakai S., Samukawa Y., Yamaguchi J.-I. (2014). A Strategy for assessing potential drug-drug interactions of a concomitant agent against a drug absorbed via an intestinal transporter in humans. Drug Metab. Dispos..

[74] Devineni D., Manitpisitkul P., Murphy J., Skee D., Wajs E., Mamidi R.N.V.S., Tian H., Vandebosch A., Wang S., Verhaeghe T. (2015). Effect of canagliflozin on the pharmacokinetics of glyburide, metformin, and simvastatin in healthy participants. Clin. Pharmacol. Drug Dev..

[75] Kasichayanula S., Liu X., Shyu W.C., Zhang W., Pfister M., Griffen S.C., Li T., LaCreta F.P., Boulton D.W. (2011). Lack of pharmacokinetic interaction between dapagliflozin, a novel sodium–glucose transporter 2 inhibitor, and metformin, pioglitazone, glimepiride or sitagliptin in healthy subjects. Diabetes Obes. Metab..

[76] Dawra V.K., Cutler D.L., Zhou S., Krishna R., Shi H., Liang Y., Alvey C., Hickman A., Saur D., Terra S.G. (2019). Assessment of the drug interaction potential of ertugliflozin with sitagliptin, metformin, glimepiride, or simvastatin in healthy subjects. Clin. Pharmacol. Drug Dev..

[77] Sasaki T., Seino Y., Fukatsu A., Ubukata M., Sakai S., Samukawa Y. (2015). Absence of Drug–Drug Interactions Between Luseogliflozin, a Sodium–Glucose Co-transporter-2 Inhibitor, and Various Oral Antidiabetic Drugs in Healthy Japanese Males. Adv. Ther..

[78] Veltkamp S.A., van Dijk J., Collins C., van Bruijnsvoort M., Kadokura T., Smulders R.A. (2012). Combination treatment with ipragliflozin and metformin: A randomized, double-blind, placebo-controlled study in patients with type 2 diabetes mellitus. Clin. Ther..

[79] Kasahara N., Fukase H., Ohba Y., Saito T., Miyata K., Iida S., Takano Y., Ikeda S., Harigai M., Terao K. (2016). A pharmacokinetic/pharmacodynamic drug–drug interaction study of tofogliflozin (a new SGLT2 inhibitor) and selected anti-type 2 diabetes mellitus drugs. Drug Res..

[80] Jeong S.I., Kim Y., Nah J.J., Huh W., Jang I., Hwang J.G., Lee S. (2023). Pharmacokinetic and pharmacodynamic interaction of DWP16001, a sodium–glucose cotransporter 2 inhibitor, with gemigliptin and metformin in healthy adults. Br. J. Clin. Pharmacol..

[81] Yoon S., Park M.S., Jin B.H., Shin H., Na J., Huh W., Kim C.O. (2023). Pharmacokinetic and pharmacodynamic interaction of DWP16001, a sodium-glucose cotransporter-2 inhibitor, with phentermine in healthy subjects. Expert. Opin. Drug Metab. Toxicol..

[82] Wang L., Wu C., Shen L., Liu H., Chen Y., Liu F., Wang Y., Yang J. (2016). Evaluation of drug–drug interaction between henagliflozin, a novel sodium-glucose co-transporter 2 inhibitor, and metformin in healthy Chinese males. Xenobiotica.

[83] Smulders R.A., Zhang W., Veltkamp S.A., van Dijk J., Krauwinkel W.J.J., Keirns J., Kadokura T. (2012). No pharmacokinetic interaction between ipragliflozin and sitagliptin, pioglitazone, or glimepiride in healthy subjects. Diabetes Obes. Metab..

[84] Kinoshita S., Kondo K. (2015). Evaluation of pharmacokinetic and pharmacodynamic interactions of canagliflozin and teneligliptin in Japanese healthy male volunteers. Expert. Opin. Drug Metab. Toxicol..

[85] Vakkalagadda B., Lubin S., Reynolds L., Liang D., Marion A.S., LaCreta F., Boulton D.W. (2016). Lack of a pharmacokinetic interaction between saxagliptin and dapagliflozin in healthy subjects: A randomized crossover study. Clin. Ther..

[86] Kim D., Choi M., Jin B.H., Hong T., Kim C.O., Yoo B.W., Park M.S. (2023). Pharmacokinetic and pharmacodynamic drug–drug interactions between evogliptin and empagliflozin or dapagliflozin in healthy male volunteers. Clin. Transl. Sci..

[87] Brand T., Macha S., Mattheus M., Pinnetti S., Woerle H.J. (2012). Pharmacokinetics of empagliflozin, a sodium glucose cotransporter-2 (SGLT-2) inhibitor, coadministered with sitagliptin in healthy volunteers. Adv. Ther..

[88] Rizos C.V., Filippatos T.D., Elisaf M.S. (2018). Pharmacokinetic drug evaluation of empagliflozin plus linagliptin for the treatment of type 2 diabetes. Expert. Opin. Drug Metab. Toxicol..

[89] Kim B., Huh K.Y., Hwang J.G., Nah J., Huh W., Cho J.M., Jang I., Yu K., Kim Y., Lee S. (2023). Pharmacokinetic and pharmacodynamic interaction between DWP16001, an sodium–glucose cotransporter 2 inhibitor and metformin in healthy subjects. Br. J. Clin. Pharmacol..

[90] Macha S., Mattheus M., Pinnetti S., Broedl U.C., Woerle H.J. (2015). Pharmacokinetics of empagliflozin and pioglitazone after coadministration in healthy volunteers. Clin. Ther..

[91] Jang K., Jeon J.-Y., Moon S.J., Kim M.-G. (2020). Evaluation of the pharmacokinetic interaction between lobeglitazone and dapagliflozin at steady state. Clin. Ther..

[92] Kim H., Kim C.O., Park H., Park M.S., Kim D., Hong T., Shin Y., Jin B.H. (2023). Evaluation of pharmacokinetic interactions between lobeglitazone, empagliflozin, and metformin in healthy subjects. Transl. Clin. Pharmacol..

[93] Kim Y.K., Hwang J.G., Park M.K. (2021). No Relevant Pharmacokinetic Drug–Drug Interaction Between the Sodium-Glucose Co-Transporter-2 Inhibitor Empagliflozin and Lobeglitazone, a Peroxisome Proliferator-Activated Receptor-γ Agonist, in Healthy Subjects. Drug Des. Dev. Ther..

[94] Que L., Huang K., Xiang X., Ding Y., Chu N., He Q. (2022). No apparent pharmacokinetic interactions were found between henagliflozin: A novel sodium-glucose co-transporter 2 inhibitor and glimepiride in healthy Chinese male subjects. J. Clin. Pharm. Ther..

[95] Nakajo I., Taniuchi Y., Yoshida S., Kadokura T., Kageyama S. (2012). Drug interaction study of ipragliflozin and migitol in healthy japanese subjects. Clin. Pharmacol. Ther..

[96] Macha S., Koenen R., Sennewald R., Schöne K., Hummel N., Riedmaier S., Woerle H.J., Salsali A., Broedl U.C. (2014). Effect of gemfibrozil, rifampicin, or probenecid on the pharmacokinetics of the SGLT2 inhibitor empagliflozin in healthy volunteers. Clin. Ther..

[97] Imamura A., Kusunoki M., Ueda S., Hayashi N., Imai Y. (2013). Impact of voglibose on the pharmacokinetics of dapagliflozin in Japanese patients with type 2 diabetes. Diabetes Ther..

[98] Dyavar S.R., Mykris T.M., Winchester L.C., Scarsi K.K., Fletcher C.V., Podany A.T. (2020). Hepatocytic transcriptional signatures predict comparative drug interaction potential of rifamycin antibiotics. Sci. Rep..

[99] Devineni D., Vaccaro N., Murphy J., Curtin C., Mamidi R.N., Weiner S., Wang S.-S., Ariyawansa J., Stieltjes H., Wajs E. (2015). Effects of rifampin, cyclosporine A, and probenecid on the pharmacokinetic profile of canagliflozin, a sodium glucose co-transporter 2 inhibitor, in healthy participants. Int. J. Clin. Pharmacol. Ther..

[100] Kasichayanula S., Liu X., Griffen S.C., Lacreta F.P., Boulton D.W. (2013). Effects of rifampin and mefenamic acid on the pharmacokinetics and pharmacodynamics of dapagliflozin. Diabetes Obes. Metab..

[101] Dawra V.K., Sahasrabudhe V., Liang Y., Matschke K., Shi H., Hickman A., Saur D., Terra S.G., Cutler D.L. (2018). Effect of rifampin on the pharmacokinetics of ertugliflozin in healthy subjects. Clin. Ther..

[102] Kasichayanula S., Chang M., Liu X., Shyu W.-C., Griffen S.C., LaCreta F.P., Boulton D.W. (2012). Lack of pharmacokinetic interactions between dapagliflozin and simvastatin, valsartan, warfarin, or digoxin. Adv. Ther..

[103] Macha S., Sennewald R., Rose P., Schoene K., Pinnetti S., Woerle H.J., Broedl U.C. (2013). Lack of clinically relevant drug–drug interaction between empagliflozin, a sodium glucose cotransporter 2 inhibitor, and verapamil, ramipril, or digoxin in healthy volunteers. Clin. Ther..

[104] Heise T., Mattheus M., Woerle H.J., Broedl U.C., Macha S. (2015). Assessing pharmacokinetic interactions between the sodium glucose cotransporter 2 inhibitor empagliflozin and hydrochlorothiazide or torasemide in patients with type 2 diabetes mellitus: A randomized, open-label, crossover study. Clin. Ther..

[105] Wilcox C.S., Shen W., Boulton D.W., Leslie B.R., Griffen S.C. (2018). Interaction between the sodium-glucose–linked transporter 2 inhibitor dapagliflozin and the loop diuretic bumetanide in normal human subjects. J. Am. Heart Assoc..

[106] Ali A.B.H., Abdel-aal F.A.M., Rageh A.H., Mohamed A.I. (2022). Cytochrome P450 3A4-mediated pharmacokinetic interaction study between tadalafil and canagliflozin using high-performance thin-layer chromatography. J. Sep. Sci..

[107] Devineni D., Vaccaro N., Polidori D., Rusch S., Wajs E. (2014). Effects of hydrochlorothiazide on the pharmacokinetics, pharmacodynamics, and tolerability of canagliflozin, a sodium glucose co-transporter 2 inhibitor, in healthy participants. Clin. Ther..

[108] Macha S., Rose P., Mattheus M., Pinnetti S., Woerle H.J. (2013). Lack of drug–drug interaction between empagliflozin, a sodium glucose cotransporter 2 inhibitor, and warfarin in healthy volunteers. Diabetes Obes. Metab..

[109] Macha S., Mattheus M., Pinnetti S., Woerle H.J., Broedl U.C. (2013). Effect of empagliflozin on the steady-state pharmacokinetics of ethinylestradiol and levonorgestrel in healthy female volunteers. Clin. Drug Investig..

[110] He X., Liu G., Chen X., Wang Y., Liu R., Wang C., Huang Y., Shen J., Jia Y. (2023). Pharmacokinetic and pharmacodynamic interactions between henagliflozin, a novel selective SGLT-2 inhibitor, and warfarin in healthy Chinese subjects. Clin. Ther..

[111] Chen Q., Yu C., Wu Q., Song R., Liu Y., Feng S., Yu C., Jia J. (2024). Evaluation of Drug-Drug Interaction Between Henagliflozin and Hydrochlorothiazide in Healthy Chinese Volunteers. Drug Des. Dev. Ther..

[112] Huang Y., Liu R., Wang Y., Liu G., Wang C., Chen X., Jia Y., Shen J. (2022). Evaluation of pharmacokinetic interactions between the new SGLT2 inhibitor SHR3824 and valsartan in healthy Chinese volunteers. Clin. Ther..

[113] Chen C., Cai D., Winnett C.E., Verma N., Preciado P. (2022). Effect of Multiple Doses of Sparsentan on the Single-Dose Pharmacokinetics of Dapagliflozin: Open-Label Drug-Drug Interaction Study in Healthy Adults: FR-PO217. J. Am. Soc. Nephrol..

[114] Stöllberger C., Finsterer J., Schneider B. (2023). Adverse events and drug-drug interactions of sodium glucose co-transporter 2 inhibitors in patients treated for heart failure. Expert. Rev. Cardiovasc. Ther..

[115] Gao F., Hall S., Bach L.A. (2020). Myopathy secondary to empagliflozin therapy in type 2 diabetes. Endocrinol. Diabetes Metab. Case Reports.

[116] Brailovski E., Kim R.B., Juurlink D. (2020). Rosuvastatin Myotoxicity After Starting Canagliflozin Treatment: A Case Report. Ann. Intern. Med..

[117] Yoshioka H., Ohishi R., Hirose Y., Torii-Goto A., Park S.J., Miura N., Yoshikawa M. (2019). Chronopharmacology of dapagliflozin-induced antihyperglycemic effects in C57BL/6J mice. Obes. Res. Clin. Pract..

[118] Wang D., Liu J., Zhou L., Zhang Q., Li M., Xiao X. (2022). Effects of Oral Glucose-Lowering Agents on Gut Microbiota and Microbial Metabolites. Front. Endocrinol..

